# Unraveling the Role of Water in Isothermal Methanol Partial Oxidation to Methyl Formate on Gold: A Combined Experimental and Computational Study

**DOI:** 10.1021/acs.jpcc.4c05968

**Published:** 2025-01-10

**Authors:** S. Eltayeb, L. L. Carroll, J. M. Correa-Hoyos, C. D. Feldt, B. Switon, W. Riedel, L. V. Moskaleva, T. Risse

**Affiliations:** a Institut für Chemie und Biochemie, Freie Universität Berlin, Arnimallee 22, 14195 Berlin, Germany; b Department of Chemistry, Faculty of Natural and Agricultural Sciences, University of the Free State, PO Box 339, Bloemfontein 9300, South Africa; c Institute of Fundamental Physics, Consejo Superior de Investigaciones Científicas, Madrid E-28006, Spain

## Abstract

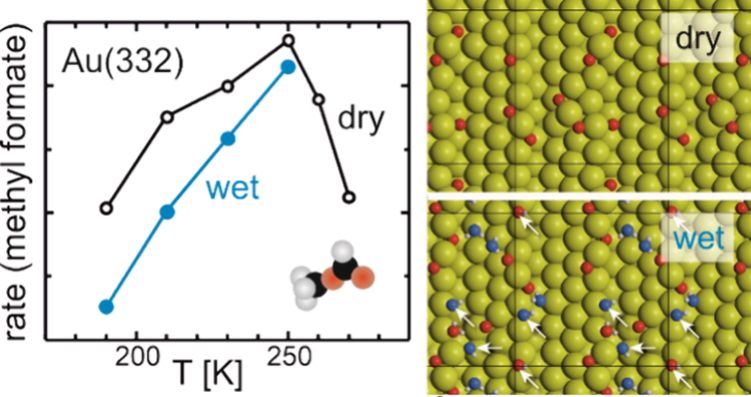

Since water is both a product and a common reactant impurity in the (partial) methanol oxidation to methyl formate (MeFo) on gold, its effect on the isothermal selectivity to methyl formate was investigated under well-defined single-collision conditions employing pulsed molecular beam experiments and in situ IRAS measurements. Both a flat Au(111) and a stepped Au(332) surface were used as model catalysts to elucidate how water affects the reactivity of low-coordinated step sites as compared to (111) terrace sites employing a range of reaction conditions. The interactions of water with methanol/methoxy as well as with oxygen species are addressed. Theoretical calculations, including static DFT and *ab initio* molecular dynamics (AIMD) simulations, are employed to enhance the microscopic understanding of water-induced changes in the oxygen species and overoxidation reactivity on gold, which are essential for the selectivity. The results provide not only information on conditions that mitigate the generally negative effect of water on methyl formate formation but also atomic-level insights into water-induced changes in the complex reaction network that governs the reactivity in applied gold catalysts, such as nanoporous gold.

## Introduction

Methyl formate (MeFo) is an important precursor in the production of a number of bulk chemicals, such as formic acid or (dimethyl)formamide, and has recently been proposed as a fuel (additive) as well as a potential hydrogen storage molecule.^1−5^ Industrially, it is currently produced by the reaction of methanol with CO over alkali metal methoxide catalysts, which require water and CO_2_-free feeds.^2,3,6^ To minimize the use of toxic reactants and to lower the purity requirements on the reactants, alternative synthesis routes are being explored, including the aerobic partial oxidation of methanol.^1,2^ For the formation of MeFo from the aerobic partial oxidation of methanol, nanoporous gold (npAu) is a promising catalyst exhibiting a high MeFo selectivity at high conversion even at temperatures below 100 °C.^7^ Nanoporous gold is prepared by etching a less noble metal (e.g., Ag) from an alloy with gold. This results in a porous ligament structure with a large number of low-coordinated sites as well as extended terraces.^8^ It consists mainly of gold but also contains residual amounts of the less noble metal, which is important for the activation of molecular oxygen in aerobic oxidations.^7,9^

For industrial applications, a process that allows for the use of water-containing (methanol) feeds, i.e., lowering the purity requirements for the feeds, would be highly desirable. Since water is a reaction product of methanol oxidation and consequently the water content increases with increasing catalytic conversion, the high selectivity of npAu toward MeFo at high conversion in gas-phase methanol oxidation suggests that water does not (negatively) affect the activity of the npAu catalyst.^7^ For CO oxidation over npAu, the addition of water was even found to be beneficial, which was attributed to an enhanced mobility of oxygen in Au_*x*_O_*y*_ islands due to the formation of hydroxyl (OH) species with water.^10−14^ However, computational studies suggest that water can react with activated oxygen species on the surface to form OH groups, which often exhibit higher barriers compared to (individual) oxygen atoms for several of the reaction steps involved in MeFo formation, potentially reducing the MeFo rate.^11,15−20^ In line with this, it has been reported for liquid phase methanol oxidation over npAu that even relatively small amounts of water in the feed are detrimental to MeFo selectivity, which has been tentatively attributed to diol formation.^21^ Surprisingly, this negative effect of water was observed even at water contents lower than those expected for high conversion in the gas phase. These seemingly contradictory results indicate that the role of water in oxidations over npAu catalysts is complex. However, obtaining a microscopic understanding of the surface processes for applied npAu studies is complicated by multiple collision conditions that allow for subsequent reactions of products, by varying reactant and product concentrations across the reactor bed to increase conversion, and by a variety of surface sites that may differ in reactivity.

Model studies using simplified catalysts, such as single-crystal surfaces, combined with well-defined single-collision conditions by applying ultrahigh vacuum (UHV) conditions, have proven successful in enhancing the atomic-level understanding of surface reactions. Based on model studies, a reaction mechanism for MeFo formation from methanol oxidation over Au surfaces has been proposed:^22−24^ In a first step, the O–H bond in methanol is selectively activated by surface-adsorbed activated oxygen, which acts as a Bro̷nsted base to form adsorbed methoxy species. Note that molecular oxygen does not dissociate under UHV conditions requiring the use of activated oxygen, such as atomic oxygen or ozone in these model studies, which may also form accumulated Au_*x*_O_*y*_ phases on the gold surface.^10,25^ Subsequent dehydrogenation of methoxy by another oxygen leads to the formation of formaldehyde, which is considered to be the rate-limiting step in the presence of activated oxygen. The formation of MeFo involves a reaction of formaldehyde with an adsorbed methoxy species to form a hemiacetal that undergoes oxidative dehydrogenation, resulting in MeFo. This coupling pathway competes with other reactions. In particular, formaldehyde can desorb before reacting with a methoxy, limiting MeFo formation under single-collision conditions. On npAu, short contact times with the catalyst bed lead to a similar effect. In addition to desorption, formaldehyde may undergo undesired overoxidation via a formate intermediate, which subsequently results in CO_2_ formation. All the steps in the reaction mechanism can also proceed with OH instead of activated oxygen (atoms) exhibiting, however, different activation barriers.^17^ In the presence of (large amounts of) OH, additional competing pathways may also occur, including the formation of the methyl diol, as suggested for liquid phase methanol oxidation on npAu in the presence of added water.^21^

While model studies have elucidated some important aspects of methanol oxidation on Au surfaces, the role of water has not yet been understood. One reason for this is that the effect of water may not be detectable in the more commonly applied temperature-programmed reaction (TPR) measurements, since water desorbs below the formation temperature of methyl formate. In contrast, isothermal pulsed molecular beam experiments are expected to be well suited to address this question, as the surface is exposed to the reactants at a surface temperature high enough for the reaction to occur. Moreover, these MB experiments allow for a more direct comparison with applied npAu studies, which are also performed under isothermal conditions.

To unravel the role of water on the reaction network in the methanol oxidation on Au, isothermal pulsed MB experiments are used to compare the kinetics in the presence and absence of added water. The experiments were carried out on a flat Au(111) surface as well as on a stepped Au(332) surface, which exhibits six-atom-wide (111) terraces as well as a large number of low-coordinated (step) sites. Thereby, the influence of different surface sites under water-rich conditions is also addressed. Moreover, the effect of water has been studied for a range of reaction conditions varying the methanol and oxygen fluxes as well as the surface temperature. For all the experiments performed in the presence of water, a high, constant water flux was chosen to emulate high conversion and/or a high level of water impurities in the methanol. This allows us to investigate the interaction of water with methanol (or methoxy) as well as with oxygen species adsorbed on the surfaces. In addition, static DFT calculations and ab initio molecular dynamics (AIMD) simulations were performed to improve the microscopic understanding of how water affects the interaction of adsorbed oxygen with the gold surface.

## Experimental Procedures

The measurements were carried out in a UHV apparatus consisting of two chambers maintained at a base pressure of 1 × 10^–10^ mbar, which has been described in detail previously.^26^ The preparation chamber contains a sputter gun (IQE 11/35, Specs) used for sample cleaning by Ar^+^ ion bombardment and a low-energy electron diffraction (LEED) system (Omicron MCP LEED) to investigate the long-range order of the sample surface. Moreover, the preparation chamber is equipped for TPD measurements with a quadrupole mass spectrometer (Prisma, Pfeiffer) featuring a Feulner cup used to minimize undesired signals due to desorption from, e.g., the sample holder. The scattering chamber is equipped with two effusive molecular beams (MBs)^27^ for sample exposure to molecular reactants, such as methanol and water, a thermal oxygen cracker (Dr. Eberl MBE-Komponenten GmbH) that provides a well-defined flux of oxygen atoms by using a pinhole doser to control the oxygen flux through the cracker, and a stagnation flow monitor with a high-precision ion gauge (360 Stabil-Ion, Granville-Phillips) to measure the pressure of the effusive beam sources at the sample position as a function of the inlet pressure of the effusive MB. The temporal evolution of the gas-phase product MeFo during pulsed isothermal MB experiments (H_3_COCHO^+^ at *m*/*z* = 60) is characterized by gas-phase quadrupole mass spectrometric (QMS) analysis (MAX-500HT, Extrel). In situ IR spectroscopy (IRAS) (IFS 66v, Bruker) (256 scans, nominal resolution of 4 cm^–1^, zero filling factor of 16) was used to provide information on the surface species present during the reaction.

The Au single crystals (10 mm diameter, 2 mm thick, Mateck) were pressed by Mo clamps onto a boron nitride heater (HT-01, Momentive), which is attached to a homemade Mo holder that is connected to a liquid nitrogen-cooled Cu block allowing for sample cooling down to approximately 100 K. The crystal temperature is measured by a type K thermocouple inserted in a 0.2 mm hole in the Au crystal edge. A commercial PID controller (3508, Eurotherm) is used to monitor the thermocouple voltage and to control the sample temperature in the isothermal experiments. The Au crystals were cleaned by repeated cycles of Ar^+^ ion bombardment (1.5 × 10^–5^ mbar, ∼6–7 μA,1 keV) for 15 and 30 min for Au(332) and Au(111), respectively, at room temperature, followed by annealing *in vacuo* (at 1000 K for 10 min for Au(332), and for Au(111) at 900 K for 10 min and subsequently at 700 K for 30 min) until a sharp LEED image expected for the respective surface was observed.^28,29^ Methanol (Honeywell Riedel-de Haën, Chromasolv, ≥99.9%; dried over a molecular sieve, 3 Å) and H_2_O were cleaned by repeated freeze–pump–thaw cycles, and oxygen ^16^O_2_ (Air Liquide, 99.998%) was used without further purification. For all measurements, the atomic oxygen source was operated at 1620 °C (14.15 V, 15.52 A).

The opening and closing of the effusive beam and thermal cracker were automated using a custom-made LabVIEW program. The pulse sequence applied in the isothermal MB measurements consists of a constant exposure of methanol (and water) and pulse(s) of oxygen (200 s on, 300 s off). Both methanol and water were supplied in excess of the atomic oxygen flux in the gas phase. During the isothermal MB experiments, in situ IRAS spectra were acquired before, during, and after the oxygen pulse to monitor surface-adsorbed species. The IRAS measurements were started approximately 10 s after beginning or end of the oxygen pulse and had a duration of approximately 3 min. The partial pressure of MeFo (molecular ion at *m*/*z* 60) in the chamber is monitored as a function of time (1 s time resolution) using QMS with electron impact ionization (1 mA, −70 eV). To quantify the MeFo formation, the QMS signal at *m*/*z* = 60 was independently calibrated by exposing the chamber to varying pressures of MeFo.^30^ The molecular fluxes of methanol and water were calculated according to calibrations with the beam monitor using Ar. The flux of atomic oxygen was calibrated by comparing TPD measurements of oxygen on Au with TPD measurements of a well-defined amount of oxygen on Pt(111)^31,32^ using the same setup. When expressing the surface coverage of oxygen atoms in monolayers (MLs), one ML corresponds to 1.4 × 10^15^ cm^–2^ and, thus, to one O atom per Au surface atom. The MeFo selectivity was calculated with respect to the available oxygen atoms, considering that two oxygen atoms are required for MeFo formation.

## Computational Procedures

### “Static” DFT Computations

In this study, “static” density functional theory (DFT) calculations were performed to investigate reaction energy profiles on the Au(221) surface. The stepped Au(221) surface exhibits an atom arrangement similar to the experimentally used stepped Au(332), but the terraces are only three atoms wide resulting in a smaller unit cell size, which allows for shorter calculation times. The Au(221) surface was modeled using a p(4 × 1) unit cell in the lateral directions. The slab thickness was 7.7 Å, with 7.2 Å vacuum space separating the slab from its periodic image in the *z* direction. The unit cell parameters were *a* = 8.78 Å, *b* = 11.71 Å, *c* = 14.90 Å, and α = β = γ = 90°. The atoms of the bottom half of the slab were kept frozen at their bulk positions, while the remaining atoms were allowed to relax without constraints. The bulk gold positions for the bottom layers were taken from the calculated lattice constant of 4.17 Å. Au–O chains were constructed along the surface steps, with “long” chains consisting of three O atoms forming either an O–Au–O–Au–O or O–Au–OH–Au–O sequence and two linear O–Au–O or O–Au–OH fragments and with “short” chains consisting of one linear O–Au–O or O–Au–OH fragment.

The Vienna Ab initio Simulation Package (VASP) was employed for the “static” DFT calculations.^33^ We used the projector augmented wave (PAW) method and a plane-wave basis set.^34,35^ The Perdew, Burke, and Ernzerhof (PBE) exchange-correlation functional was applied.^36^ A 5 × 5 × 1 *k*-point mesh and Monkhorst–Pack grids were used for the Brillouin zone integrations.^37^ The calculations utilized a plane-wave cutoff energy of 415 eV and a kinetic energy cutoff of 645 eV. The Methfessel-Paxton order 1 smearing scheme with a smearing parameter of 0.05 eV was employed.^38^ Geometry relaxation (using the conjugate gradient method) was continued until the force acting on each atom was less than 0.02 eV/Å, and energy convergence was achieved with a tolerance of 10^–6^ eV. The minimum energy reaction paths were determined by the climbing-image nudged elastic band method (ci-NEB),^39^ and transition-state structures were further refined using the dimer method.^40^

### Ab Initio Molecular Dynamics

*Ab initio* molecular dynamics (AIMD) simulations were performed using the open-source MD simulation package CP2K, chosen for its high computational efficiency.^41^ This code was used for optimizing the initial structures and subsequent AIMD simulations. A p(2 × 6) unit cell of Au(221) with an 8 Å slab thickness and a 17 Å vacuum region separating the periodic images along the *z*-direction was utilized. The bottom layer of the structure was constrained, while the top two layers were left unconstrained. Oxygen atoms and water molecules were placed pseudo-randomly on the Au(221) surface using a Python script that incorporates a convex hull algorithm coupled with triangulation, point generation, and distance checks.

Simulations were carried out within the NVT ensemble at 700 K, employing the Nosé–Hoover thermostat. The NVT ensemble (canonical ensemble) is a statistical ensemble that is used to study a thermodynamic system under the conditions of a constant particle number *N*, constant volume *V*, and a temperature fluctuating around an equilibrium value ⟨*T*⟩. The system is allowed to exchange heat with outer space so that its temperature stays constant, while thermostats are used to control the temperature. The ensemble and thermostat selection is based on established protocols and previous research. A time step of 1 fs was selected, with MD frames printed every 20 fs. Exchange-correlation energy calculations were performed using the PBE functional alongside Goedecker–Teter–Hutter PBE pseudopotentials.^36,42,43^ Gaussian and plane-wave (GPW) basis sets were employed, with a multigrid energy cutoff of 500 Ry.^44^ CP2K’s double-ζ basis sets, optimized for the GTH pseudopotentials, were selected to avoid basis set superposition errors. Brillouin zone integration for AIMD simulations was limited to the Γ point. D3 dispersion corrections were applied in the AIMD simulations to prevent water desorption. In our previous computational work on CO oxidation,^45^ we analyzed the adsorption energies of CO and O_2_ with and without dispersion and found that D3 correction tends to overestimate the adsorption energies. Therefore, we decided not to include the dispersion correction in the static DFT calculations, but just in the AIMD simulations. The self-consistent electronic minimization convergence threshold was set at 10^–6^ eV.

Atom mobility was studied by considering the root mean squared deviation of a selection of atoms, according to the equation:
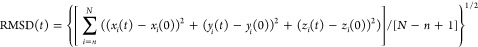
where *x*_*i*_(*t*), *y*_*i*_(*t*), and *z*_*i*_(*t*) define the position of an atom *i* at time *t*, and *x*_*i*_(0), *y*_*i*_(0), and *z*_*i*_(0) are the coordinates of an atom *i* at the initial MD frame, with *n* and *N* denoting the range of atoms considered for calculation of the RMSD.

## Results and Discussion

### Isothermal MB Experiments on the Effect of Water on MeFo Formation

To address the kinetic importance of water on the selectivity toward MeFo in methanol oxidation, isothermal MB experiments under well-defined single-collision conditions were conducted, both in the presence and absence of added water, referred to as wet and dry conditions, respectively. In all experiments, atomic oxygen was pulsed onto the sample (200 s on, 300 s off) while the sample was continuously exposed to a high flux of methanol employing a methanol excess in the gas phase (factor >10). Under wet conditions, the sample was also continuously exposed to a high water flux (160 × 10^13^ s^–1^ cm^–2^). Compared to the applied methanol fluxes, the applied water flux was significantly higher (factor of >3). This applied water flux corresponds to high methanol conversions of 75% or even 97% for the high and low methanol fluxes, respectively. The effect of water on the MeFo formation was investigated for two different fluxes of atomic oxygen and two different fluxes of methanol as well as for a range of surface temperatures. Moreover, the reaction was investigated on both a flat Au(111) surface and a stepped Au(332) surface to address differences due to the presence of low-coordinated sites.

**Figure 1:**
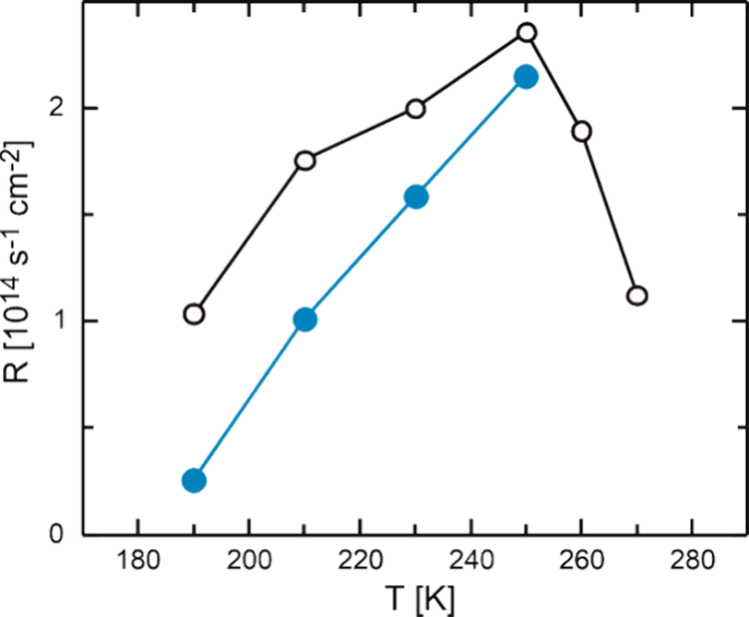
MeFo rate at the end of the oxygen pulse in isothermal MB experiments on Au(332) under dry (black) and wet (blue) conditions as a function of sample temperature, applying a constant, high flux of methanol (52.7 × 10^13^ s^–1^ cm^–2^) and pulsing a rather high flux of atomic oxygen (0.4 × 10^13^ s^–1^ cm^–2^, 200 s on, 300 s off). An additional continuous, high flux of water (1.6 × 10^15^ s^–1^cm^–2^) was applied for the measurements under wet conditions.

In Figure 1, the MeFo formation rate achieved at the end of the oxygen pulse is shown exemplarily for dry and wet conditions on the stepped Au(332) surface applying a high methanol flux (52.7 × 10^13^ s^–1^ cm^–2^) and a rather high atomic oxygen flux (0.4 × 10^13^ s^–1^ cm^–2^). For the dry conditions, the results have been presented and discussed in detail previously.^46^ Briefly, the MeFo formation rate initially increases, as the temperature is raised due to the increase in the rate constant for higher temperatures. As the temperature is further increased, the MeFo rate exhibits a maximum and decreases for even higher temperatures. This can be attributed to increased desorption of molecular reactants or intermediates, such as methanol or formaldehyde, at elevated temperatures, which lowers their transient surface concentrations and consequently also the reaction rate to MeFo. Under wet conditions, the MeFo formation rate is lowered as compared to dry conditions. The negative effect of water is more pronounced at low temperatures and decreases with increasing temperature. At higher temperatures, the increase in the rate constants of the selective pathway may reduce the negative effect of water (or OH) on the MeFo formation rate, provided this benefit is not significantly offset by an increase in the rate constants of competing reactions. While the overall impact of temperature-dependent changes in rate constant on MeFo formation relative to competing reactions is difficult to predict, the increase in the desorption rate of water will lower its transient surface concentration. Consequently, the rates of all reactions involving water will decrease, making the negative effect of water on the MeFo formation less pronounced.

**Figure 2:**
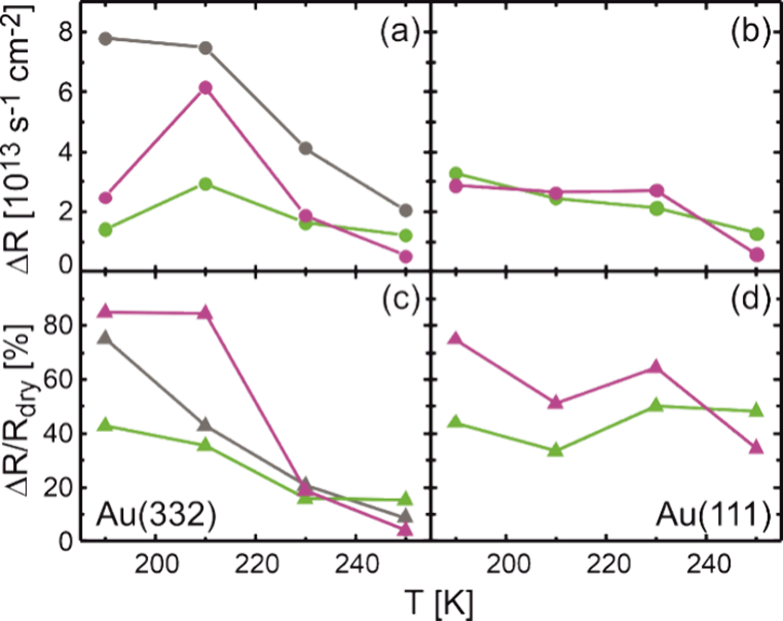
Absolute rate decrease Δ*R* = *R*_dry_ – *R*_wet_ (a, b) and relative rate decrease Δ*R*/*R*_dry_ (c, d) in methyl formate formation under wet as compared to dry conditions in isothermal pulsed MB experiments observed at the end of the oxygen pulse. The results are shown for Au(332) (a, c) and Au(111) (b, d) for different surface temperatures as well as different flux conditions. Results for a high methanol flux of 52.7 × 10^13^ s^–1^ cm^–2^ combined with a rather high atomic oxygen flux of 0.4 × 10^13^ s^–1^ cm^–2^ (gray) and a lower oxygen flux of 0.08 × 10^13^ s^–1^ cm^–2^ (green) are shown. In comparison, the results for a higher flux of atomic oxygen of 0.4 × 10^13^ s^–1^ cm^–2^ combined with a lower methanol flux of 4.3 × 10^13^ s^–1^ cm^–2^ are shown as the purple symbols.

The influence of water was also investigated for a smaller methanol flux (4.3 × 10^13^ s^–1^ cm^–2^) and a smaller atomic oxygen flux (0.08 × 10^13^ s^–1^ cm^–2^). For easy comparison of the six sets of measurements—with and without added water for three different flux conditions—Figure 2a shows the absolute rate difference Δ*R* in MeFo formation for wet and dry conditions, i.e., Δ*R*(MeFo) = *R*_dry_ – *R*_wet_, for Au(332) across the three applied flux conditions (see also Figure S1 for absolute rates). In the presence of water, the MeFo formation is also lower, when applying a lower methanol or a lower oxygen flux. This suggests that the adverse effect of water is not limited to a specific set of reaction conditions but is a more general property of the reaction system. The absolute rate decrease Δ*R* is highest when applying both high methanol and high oxygen fluxes and becomes smaller when either the methanol or the oxygen flux is decreased. This suggests that the interaction of water with both oxygen and methanol lowers the MeFo formation. However, the MeFo formation rate under dry conditions also depends on the fluxes applied and the surface temperature (see also Figure S1). To take this into account, the relative rate decrease Δ*R*/*R*_dry_ in MeFo formation is shown in Figure 2c for the different flux conditions applied on Au(332). At low temperatures, the relative rate decrease Δ*R*/*R*_dry_ is most pronounced for oxygen-rich conditions. This is consistent with the effective formation and thus high formation rates of OH species under conditions where both the oxygen and the water surface concentrations are high. Since reactions of methanol-derived intermediates with OH are often predicted by theory to be slower than those with atomic oxygen,^15−17,20,47^ the experimentally observed reduced MeFo formation rate is consistent with expectations. Moreover, the formation of OH species may affect the mobility of oxygen species in accumulated Au_*x*_O_*y*_ phases.^14^ As it was previously shown that accumulated Au_*x*_O_*y*_ phases are important for MeFo formation on Au,^46^ a changed mobility in the presence of water may also affect the MeFo formation. At higher temperatures, the relative rate change on Au(332) decreases considerably for all applied conditions (Figure 2c), but the effect is less pronounced for high methanol and low oxygen fluxes (green trace) as compared to more oxygen-rich conditions (gray and purple traces). At 250 K, the largest relative rate change is found for the methanol-rich conditions, while at lower temperatures (<230 K), the relative rate change is largest for the oxygen-rich condition (low methanol and high oxygen flux; purple trace) and, thus, reversed as compared to high temperatures. A possible explanation is that methanol and water form hydrogen bonds. This can stabilize water on the surface and thus increase the transient surface concentration of water as compared to lower methanol fluxes. Alternatively, the formation of hydrogen bonds between water (or OH) and methanol may also affect the adsorption of methanol or methoxy species, which are also capable of forming hydrogen bonds and in turn alter their transient surface concentration or reactivity. Additionally, water may lower the transient concentration of methoxy by reaction to (OH and) methanol, which desorbs quickly at elevated temperatures. The relative effect of such water–methanol interactions is expected to become kinetically more important for MeFo formation at higher temperatures, where the transient water concentration decreases more rapidly in the absence of stabilizing interactions with methanol.

In addition to the measurements on the stepped Au(332) surface, the effect of water on the methanol oxidation to MeFo was also investigated for the flat Au(111) surface. The MeFo formation rates under wet conditions are also reduced for the flat Au(111) surface, as compared to dry conditions (see Figure 2b,d and Figure S1). Similar to Au(332), the absolute rate decrease Δ*R* becomes smaller at elevated temperatures, consistent with faster water desorption and thus lower transient surface water concentrations. The influence of temperature on the effect of water is overall less pronounced for Au(111) as compared to Au(332). The relative rate decrease Δ*R*/*R*_dry_ for Au(111) at low temperatures is more pronounced for oxygen-rich conditions (purple trace) but slightly larger at higher temperatures for methanol-rich conditions (green trace), similar to the observations for the stepped Au(332) surface.

### Effect of Water Interactions with Methanol on Au(111) and Au(332)

A more detailed comparison of the MeFo formation on the two different gold surfaces shows for methanol-rich conditions (green, Figure 2) that the absolute rate difference Δ*R* is also quantitatively very similar for both surfaces (except for the lowest temperature). As the rate is the product of the (effective) rate constants and the surface concentrations of the reactants and intermediates, the observation of comparable rate differences across a range of temperatures implies that, for such a complex reaction network, these parameters change similarly for both surfaces. Consequently, this suggests that the effect of water under these conditions is similar for both surfaces and is presumably related to hydrogen bonding between methanol and water. Furthermore, the relative rate decrease Δ*R*/*R*_dry_ at low temperature is also comparable for both surfaces, consistent with the picture that the adverse effect of water on MeFo formation is not very different for the two surfaces. However, at elevated temperatures, the relative rate decrease Δ*R*/*R*_dry_ is notably smaller for Au(332). These observations are inconsistent with a simple picture that assumes only one type of reaction path for MeFo formation on both flat Au(111) and stepped Au(332), i.e., a simplified assumption that the reacting species in the presence of steps would not be altered in their reactivity, including changes in activation barrier, mobility, or local (transient) surface concentrations. The notion that the presence of steps does affect the reacting species is also in agreement with our previous study, suggesting an additional reaction pathway for effective MeFo formation associated with low-coordinated sites, such as step sites.^46^ Moreover, the results are inconsistent with the notion that all reaction pathways on both the flat and stepped surfaces are equally affected by water. A possible explanation is that on stepped Au(332), reaction pathways associated with low-coordinated sites, which become kinetically more important for MeFo formation at higher temperatures, are less affected by water than pathways that are also accessible on flat Au(111). At higher temperatures, fast desorption may, thus, strongly lower the transient concentrations of methanol or formaldehyde, which overcompensate the increase in reaction rate constant at higher temperatures resulting in an overall decrease in MeFo formation rate for increasing temperature (see also text to Figure 1). Under such conditions, low-coordinated step sites, which exhibit higher adsorption energies and can therefore maintain locally higher transient concentrations of these desorbing reactants, will have a higher relative contribution to the MeFo formation rate becoming kinetically more important at higher than at lower temperatures. Since methanol preferentially forms extended hydrogen-bonded structures along the √3-directions of the (111) surface^48^ that are not parallel to the direction of the steps on Au(332), the interaction of water with methanol is expected to be different for (extended) (111) terraces and the steps of Au(332), with methanol adsorbed at steps being less affected by interaction with water due to less favorable hydrogen-bonding geometries at steps. Under these conditions, hydrogen bonding of water (or hydroxyl species) and methanol (or methoxy) should reduce the adverse effect on MeFo formation at steps as compared to terrace sites. Low-coordinated step sites, which exhibit stronger binding of reactants, such as methanol, and thus, higher transient concentrations in isothermal MB experiments, allow MeFo formation to be more effectively maintained than on less strongly binding terrace sites at high temperatures, where the MeFo is limited by low transient concentrations of these reactants.^46^ Assuming a similar effect of water on the (111) terrace sites of Au(332) and Au(111), the absolute rate decrease Δ*R* at high temperatures is expected to be similar for both surfaces, while the relative rate decrease should be significantly smaller for stepped Au(332), where additional pathways connected to low-coordinated step sites that are kinetically especially important at high temperatures and less (negatively) affected by water allow for higher MeFo formation, as observed experimentally.

**Figure 3:**
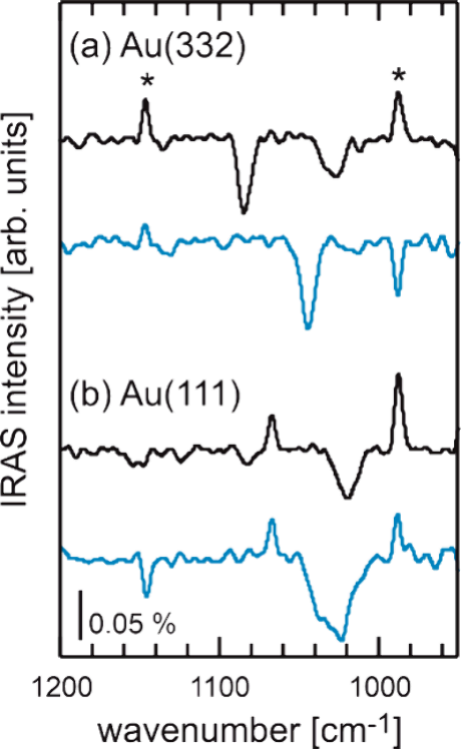
In situ IRAS measurements in the wavenumber range between 950 and 1200 cm^–1^ conducted during the isothermal, pulsed MB experiments of methanol oxidation at 190 K under dry (black) and wet (blue) conditions on (a) Au(111) and (b) Au(332). A continuous methanol flux (4.3 × 10^13^ s^–1^ cm^–2^) was applied, while atomic oxygen was pulsed (0.4 × 10^13^ s^–1^ cm^–2^, 200 s on, 300 s off). Under wet conditions, a continuous flux of water (1.6 × 10^15^ s^–1^cm^–2^) was also applied to the sample. The spectra were obtained during the oxygen pulse. The signals around 990 and 1145 cm^–1^ (indicated for the upper most spectrum with stars) are experimental artifacts.

To obtain experimental evidence for the interaction between methanol (or methoxy) and water (or OH), temperature-programmed desorption (TPD) measurements and in situ IRAS measurements were performed. The TPD measurements for methanol with and without coadsorbed water on the clean gold surfaces, i.e., in the absence of coadsorbed oxygen, showed small shifts (≤5 K) in the maximum desorption temperature of methanol, which were, however, within the experimental accuracy, thus making a conclusive interpretation of the results difficult (data not shown). In contrast, the in situ IRAS measurements shown in Figure 3 in the wavenumber range between 950 and 1200 cm^–1^ show clear differences for dry and wet conditions. The spectra were obtained during the oxygen pulse at a low surface temperature of 190 K for both the flat Au(111) surface and the stepped Au(332) surface (see also Figure S2). The low surface temperature was chosen to ensure a sufficiently high transient concentration of methanol (or methoxy) for IRAS detection. The spectra exhibit signals characteristic of surface-adsorbed methanol or methoxy.^22,30^ In the wavenumber range shown, the ν_C–O_ mode is found under dry conditions around 1030 cm^–1^ for the stepped Au(332) surface, while it is slightly red-shifted to 1020 cm^–1^ for the flat Au(111) surface. Under wet conditions, the signal maximum attributed to the ν_C–O_ mode is blue-shifted to around 1045 and 1030 cm^–1^ for Au(332) and Au(111), respectively. This spectral shift clearly indicates a change in the adsorption of methanol or methoxy in the presence of water, in agreement with the proposed hydrogen bonding between methanol/methoxy and water/hydroxyl. Looking more closely at the changes in the presence of water, it can be seen for flat Au(111) that the ν_C–O_ signal broadened significantly under wet conditions, also exhibiting intensity at the signal position observed for dry conditions. In contrast, the width of the shifted ν_C–O_ signal on the stepped Au(332) shows a similar width as under dry conditions and essentially no intensity in the range of the signal found for dry conditions. The increased signal width suggests that methanol adsorption on the planar Au(111) is rather heterogeneously affected by water. Under dry conditions, the spectrum of stepped Au(332) shows another signal around 1085 cm^–1^, which can be tentatively attributed to the CH_3_ rocking mode of methanol/methoxy. This signal is not observed for flat Au(111) and is therefore specific to methanol/methoxy adsorption in the presence of low-coordinated step sites. In the presence of water, this signal around 1085 cm^–1^ is not detected in the IRAS measurements on Au(332). This observation implies that the presence of water also modifies the adsorption of methanol/methoxy at low-coordinated step sites. In terms of the MeFo formation, these results first demonstrate that water affects the adsorption of methanol/methoxy, as suggested by the differences in reactivity under dry and wet conditions observed for both Au(111) and Au(332). Second, the IRAS results support the notion that there is an additional pathway for MeFo formation that is specific to the stepped Au(332) surface and that is affected differently by water than those accessible on Au(111).

### Effect of Water Interactions with Oxygen Species in Isothermal MB Experiments

**Figure 4:**
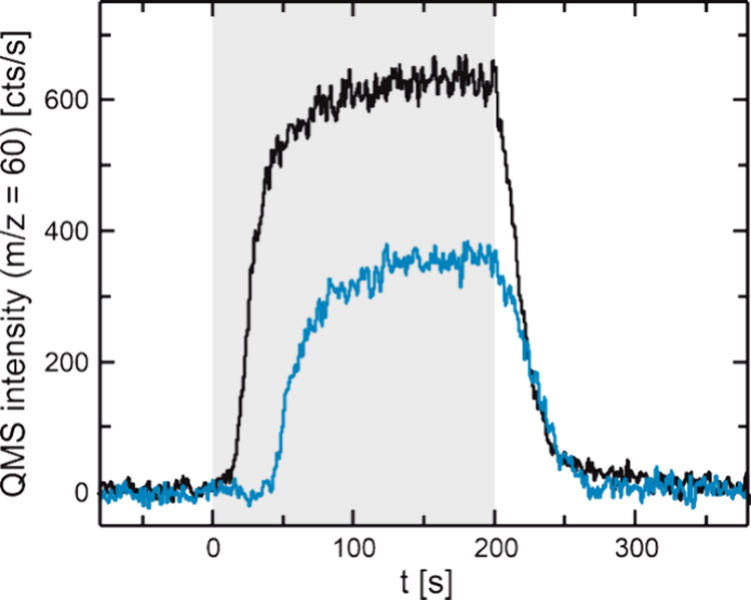
Transient MeFo formation rate (*m*/*z* = 60) in isothermal pulsed MB experiments of the methanol oxidation under dry (black) and wet (blue) conditions on Au(111) at 190 K. The sample was continuously exposed to a high flux of methanol (52.7 × 10^13^ s^–1^ cm^–2^), while a low flux of atomic oxygen was pulsed onto the sample (0.08 × 10^13^ s^–1^ cm^–2^, 200 s on, 300 s off, oxygen exposure indicated by gray box). Under wet conditions, the sample was also exposed to a continuous, high flux of water (1.6 × 10^15^ s^–1^cm^–2^).

Previous evidence has shown that Au_*x*_O_*y*_ phases are important for MeFo formation on gold surfaces.^46^ Additionally, water is suggested to influence the mobility of oxygen species within these Au_*x*_O_*y*_ phases. This raises the question of how this interaction with water impacts the MeFo formation. The effect of water on the Au_*x*_O_*y*_ phases is evident when considering the transient kinetics of the MeFo formation from the pulsed isothermal MB experiments. For example, Figure 4 illustrates the MeFo formation under methanol-rich conditions on Au(111) at a low surface temperature of 190 K. Under these conditions, the MeFo formation exhibits an induction period, i.e., (almost) no MeFo formation is observed at the beginning of the oxygen pulse, while the MeFo rate increases steeply at the end of the induction period. This induction period has previously been attributed to the formation of accumulated Au_*x*_O_*y*_ phases, which are important for MeFo formation, while (single) oxygen atoms, which are available immediately upon oxygen exposure, clearly do not allow for efficient MeFo formation under these conditions.^46^ At low surface temperatures and low oxygen fluxes, the formation of such accumulated Au_*x*_O_*y*_ phases is expected to be rather slow, allowing the induction period to be observed, while at higher temperatures or higher oxygen fluxes, the formation of these phases becomes faster, leading to an earlier onset of MeFo formation.^46^ In the presence of added water, the induction period is significantly prolonged compared to the dry conditions. This observation suggests that water affects the formation of the accumulated Au_*x*_O_*y*_ phases, thereby delaying the onset of MeFo formation. In the presence of water, the induction period is slightly less than 50 s, which is similar to and only slightly shorter than the induction period under dry conditions applying half the oxygen flux, i.e., 0.04 × 10^13^ s^–1^ cm^–2^.^46^ This suggests that only about 50% of the oxygen atoms effectively contribute to Au_*x*_O_*y*_ phase formation under the applied water flux. Moreover, the MeFo formation rate at the end of the oxygen pulse is also comparable to the MeFo formation rate at dry conditions and half the oxygen flux (0.04 × 10^13^ s^–1^ cm^–2^),^46^ consistent with the picture that only about 50% of Au_*x*_O_*y*_ phases are active for MeFo formation when a high water flux is applied. Thus, the transient kinetics of MeFo formation clearly indicate that water affects the (accumulated) oxygen species on the gold surface, thereby reducing MeFo formation.

Next, the effect of water on the MeFo formation is considered in more detail for oxygen-rich conditions. At high temperatures, the absolute rate decrease Δ*R* due to water is comparable for both surfaces, while the relative rate decrease Δ*R*/R_dry_ is notably smaller for Au(332) (see Figure 2). This is similar to high temperatures under methanol-rich conditions and is consistent with the notion of an additional pathway for MeFo formation specific to low-coordinated sites on the stepped surface, which is less affected by water at high temperatures. However, at low temperatures, both the absolute and relative rate decreases (Δ*R* and Δ*R*/*R*_dry_) are smaller on Au(111) under oxygen-rich conditions, whereas they were found to be similar for both surfaces under methanol-rich conditions. To explain this observation, an additional effect in MeFo formation is required that is specific to the stepped Au(332) surface, which is active for MeFo formation under dry conditions at low temperatures and is particularly affected by water at low temperatures. In contrast, the Au(332)-specific effect demonstrated under methanol-rich conditions at high temperatures, which has been attributed to hydrogen-bonding between methanol and water, does not contribute significantly to MeFo formation at low temperatures. A possible explanation for the additional, kinetically relevant effect for MeFo formation under oxygen-rich conditions may be related to accumulated Au_*x*_O_*y*_ phases. These are important for MeFo formation and should be more strongly affected by water at low temperatures where the transient water concentration is higher due to slower desorption, as compared to high temperatures. It is important to note that the reaction of water with activated oxygen was shown to proceed on Au(111) already at 77 K,^11,12^ suggesting that the rate of this reaction in the isothermal MB experiments conducted above the water desorption temperature is significantly affected by rather low transient water concentrations. Furthermore, Au_*x*_O_*y*_ phases on flat Au(111) and stepped Au(332) have previously been shown to differ in reactivity for methanol oxidation.^46,49^ Hence, modification of such surface-specific Au_*x*_O_*y*_ phases by water may cause the observed differences in MeFo reactivity by quenching the effective MeFo formation pathways specific to the stepped surface at low temperature.

### Effect of Water under Oxygen-Rich Conditions in MB Experiments with an Extended Pulse Sequence

**Figure 5:**
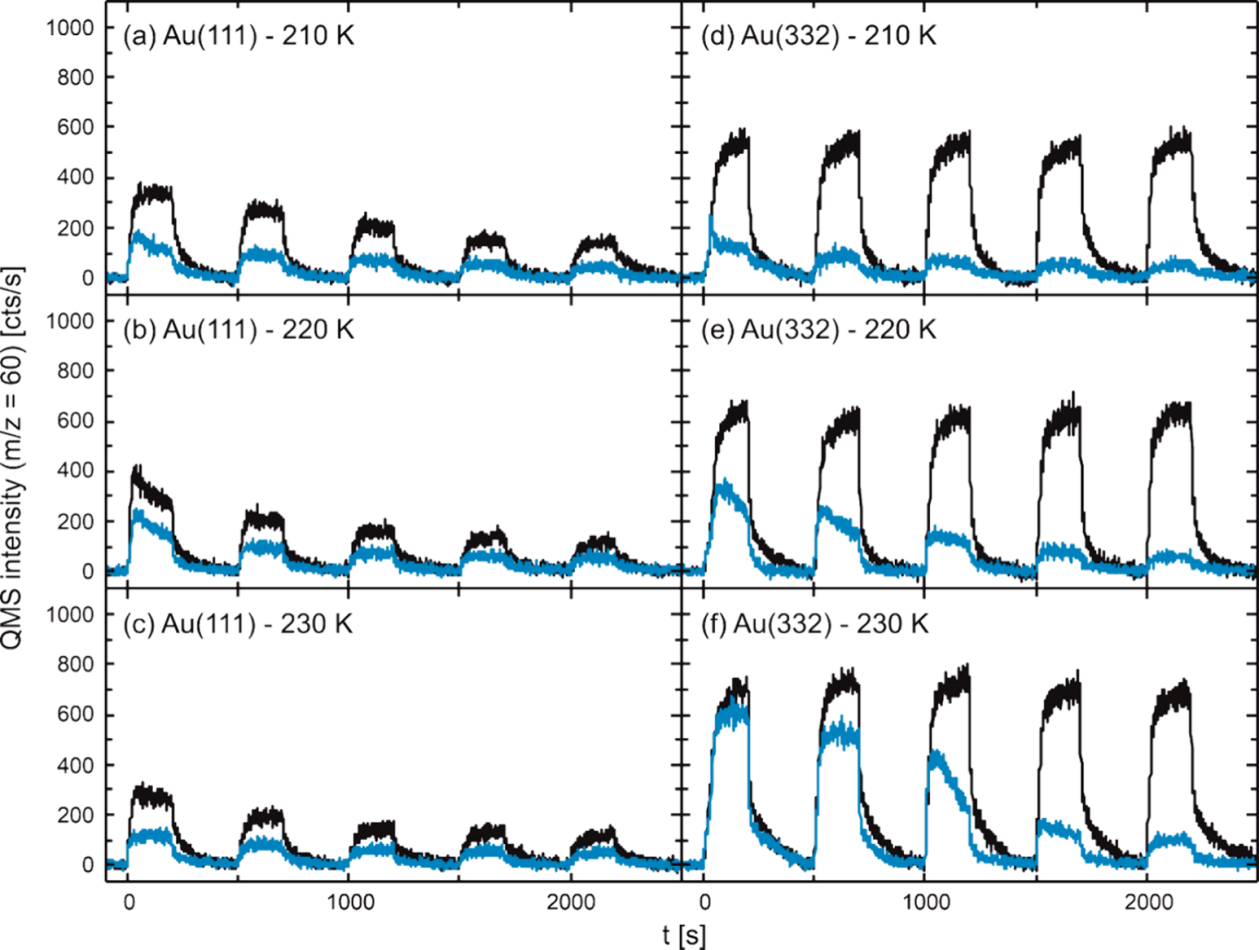
MeFo formation rate (*m*/*z* = 60) under dry (black) and wet (blue) conditions in pulsed isothermal molecular beam experiments on the methanol oxidation on (a–c) Au(111) and (d–f) Au(332) at 210 K (a, d), 220 K (b, e), and 230 K (c, f). In the experiments, a continuous, rather low flux of methanol (4.3 × 10^13^ s^–1^ cm^–2^) was applied and a rather high flux of atomic oxygen was pulsed (0.4 × 10^13^ s^–1^ cm^–2^, 5 pulses, 200 s on, 300 s off).^49^ Under wet conditions, the sample was additionally exposed to continuous, high flux of water (1.6 × 10^15^ s^–1^ cm^–2^).

To investigate how the presence of added water affects the long-term activity of the gold surfaces, isothermal MB experiments were performed applying a longer sequence of five oxygen pulses, while monitoring the MeFo formation rate. Figure 5 shows the results for Au(111) and Au(332) employing a rather low continuous methanol flux (4.3 × 10^13^ s^–1^ cm^–2^) and pulsing a rather high oxygen flux (0.4 × 10^13^ s^–1^ cm^–2^). The experiments were performed both in the absence and in the presence of a high, continuous water flux (1.6 × 10^15^ s^–1^ cm^–2^) at different surface temperatures. Under dry conditions, the MeFo formation rate on flat Au(111) decreases significantly over the pulse sequence, especially at higher temperatures. In contrast, MeFo formation on stepped Au(332) remains largely constant over the pulse sequence under these rather oxygen-rich conditions (less than 10% reduction in MeFo yield). This observation was previously attributed to a significant accumulation of formate species blocking active sites on the Au(111) surface, while a lower formation of unwanted formate species on Au(332) was associated with less active AuO chains forming at the steps of the Au(332) surface.^49^

**Figure 6:**
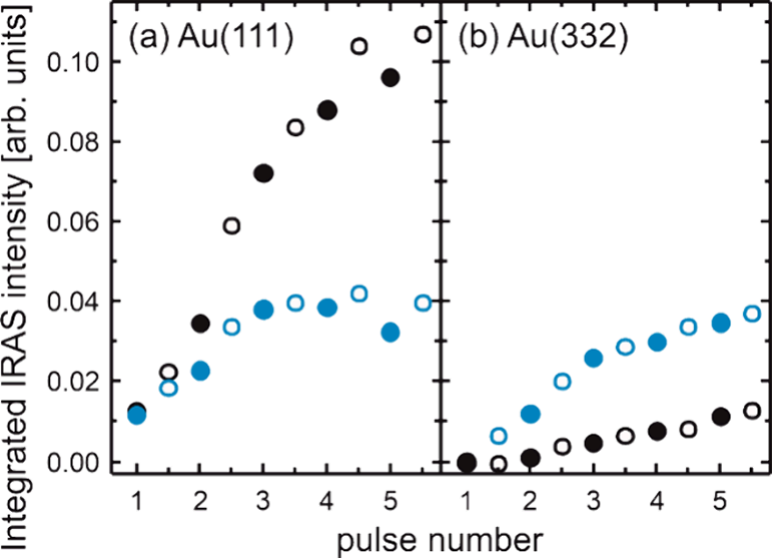
Integrated IRAS intensity of the ν_s_(OCO) stretching band of formate detected around 1330 cm^–1^ for (a) Au(111) and (b) Au(332) under dry (black) and wet (blue) conditions. The measurements were conducted at 230 K applying a methanol flux of 4.3 × 10^13^ s^–1^ cm^–2^ and a flux of atomic oxygen of 0.4 × 10^13^ s^–1^ cm^–2^, as well as a water flux of 1.6 × 10^15^ s^–1^ cm^–2^ under wet conditions (see also Figure 5c, f). The in situ IRAS measurements were conducted during (filled symbols) and between (open symbols) the oxygen pulses.

Under wet conditions, the MeFo formation rate is lowered on Au(111) compared to dry conditions. Across the pulse sequence, the MeFo formation on Au(111) decreases under wet conditions qualitatively similar to the dry conditions where the surface deactivation was associated with formate accumulation. The absolute MeFo rate decrease between dry and wet conditions becomes smaller over the pulse sequence, while the relative rate decrease remains nearly constant (see also Figure S3). The evolution of the integrated intensity of the ν_s_(OCO) stretching band of formate detected around 1330 cm^–1^ in in situ IRAS measurements for a surface temperature of 230 K is shown as an example in Figure 6a.^22,50^ In the presence of water, the formate intensity is smaller on Au(111), suggesting a lower amount of accumulated formate species on the surface under wet conditions. Assuming, based on theoretical calculations, higher activation barriers for methanol conversion to formaldehyde with OH as H-abstracting base than with oxygen species,^17^ less formate formation can be expected in the presence of water, even though similar barriers for the reaction steps from formaldehyde to formate have been calculated for OH and oxygen.^17^ In the presence of significant amounts of OH, additional overoxidation pathways to, e.g., formic acid or methanediol (formaldehyde monohydrate), may become kinetically important, which could also lower formate formation and thus its accumulation on the surface. Yet, no clear evidence for significant formation of the diol could be found in the isothermal MB experiments. In a simple site-blocking model, a reduced formate coverage should be associated with less deactivation with respect to MeFo over the pulse sequence. However, the relative rate decrease in the presence of water remains approximately constant, which is inconsistent with such a simple picture. Moreover, the MeFo formation rate under dry conditions remains nearly constant for the last three O pulses, while the intensity of the formate band more than doubles in the IRAS spectra. This suggests that some of the formate species detected by IRAS are spectator species that do not strongly affect MeFo formation.

The stepped Au(332) surface shows almost no deactivation (<10%) for MeFo formation under dry conditions for the three temperatures studied here, which is in striking contrast to the behavior in the presence of added water (Figure 5). At low temperatures, the MeFo rate is strongly suppressed compared to the dry case already at the beginning of the pulse sequence and decreases further over the pulse sequence. At 230 K, the overall deviation in MeFo formation between dry and wet conditions is small at the end of the first O pulse and increases only slightly for the second O pulse. However, the MeFo rate decreases sharply during the third O pulse. A decrease in the MeFo rate is also observed during the fourth O pulse, before reaching a low quasi-steady-state rate during the last O pulse, corresponding to about 10% of the initial MeFo rate at the beginning of the pulse sequence. The integrated IRAS intensity of the formate signal at 230 K is significantly increased for Au(332) in the presence of water, reaching an intensity similar to that observed for Au(111) under wet conditions (Figure 6). Assuming no strong changes in the adsorption geometry of formate, this suggests an increased formate accumulation on the stepped surface in the presence of water. The increased formate accumulation on stepped Au(332) under wet conditions qualitatively agrees with the MeFo rate decrease over the pulse sequence under these wet conditions. Thus, surface blocking by formate may contribute to the decrease in MeFo formation rate. However, a more detailed analysis shows that a simple picture in which all formate species contribute equally to surface deactivation for MeFo formation is inconsistent with the experimental results. First, the formate IRAS intensity under dry conditions is about half of that under wet conditions. However, the MeFo rate decreases by less than 10% over the pulse sequence in the dry case, but by about 90% in the wet case. Second, under wet conditions, the increase in formate intensity from the first to the second oxygen pulse is comparable to the increase from the second to the third oxygen pulse. However, the decrease in MeFo formation rate from the first to the second O pulse is only about 15%, but about 60% from the end of the second to the end of the third O pulse. In line with the interpretation given for Au(111), the observations are consistent with a fraction of the formate species on stepped Au(332) being spectator species that do not significantly influence the MeFo formation, while another fraction, which is notably formed only under wet conditions, reduces MeFo formation considerably. In support of this picture, a slight blue shift in the maximum of the ν_s_(OCO) stretching band of formate is detected after the second O pulse under wet conditions for Au(332), which is consistent with the formation of a different type of formate that may reduce MeFo formation more effectively than the formate previously accumulated on the surface (see Figure S4).

The increased accumulation of formate under wet conditions for the stepped Au(332) surface is rather surprising. Higher activation barriers for formaldehyde formation through reactions with OH, compared to oxygen, and potential additional overoxidation pathways to, e.g., formic acid or the diol, are both expected to decrease the availability of the precursor formaldehyde for formate formation and thus, the rate of formate formation, as observed for Au(111). However, this contrasts with the experimental results for stepped Au(332). Again, this apparent contradiction can be attributed to the formation of surface-specific Au_*x*_O_*y*_ phases on Au(322). It has been suggested that the formation of AuO chains at the steps is key to the decreased overoxidation on the stepped surface compared to the flat Au(111) under dry conditions.^49^ Specifically, previous AIMD simulations indicated the formation of extended AuO chains in the presence of steps, suggesting that most of the oxygen on a stepped surface is located within these AuO chains.^49^ While rather low activation barriers were calculated for individual oxygen atoms or oxygen at the ends of AuO chains on a stepped gold surface, oxygens in the middle of AuO chains were found to have significantly higher barriers to formate formation. Thus, the low formate formation on the stepped surface was attributed to the low availability of reactive oxygen, i.e., single O atoms or terminal oxygens at the ends of long AuO chains. Consequently, the unexpected increase in formate accumulation on Au(332) under wet conditions could be related to water-induced changes in these AuO chains. We see two possible reasons for this. First, the increase in formate formation could be related to an increased reactivity of the rather unreactive oxygen in the middle of the AuO chains upon hydroxylation, if indeed such hydroxylation occurs to some extent in the presence of water. Second, slower chain growth and thus increased availability of more reactive oxygen, such as single O atoms or oxygen at the end of AuO chains, could also enhance the overoxidation to formate. These water-induced changes of AuO chains and their possible effect on the overoxidation pathways have been modeled in the computational part of this study. Furthermore, DFT calculations show that the presence of OH species on the surface opens up additional pathways for formaldehyde overoxidation to formate via a singly protonated diolate intermediate, OCH_2_OH. The identified pathways have lower activation energies than the main CH_2_O → OCH_2_O → OCHO pathway followed under dry conditions,^49^ for which long AuO chains at the steps significantly raise the overall barrier.

### Computational Modeling of Overoxidation on the Au(221) Surface Modified by Water

**Figure 7:**
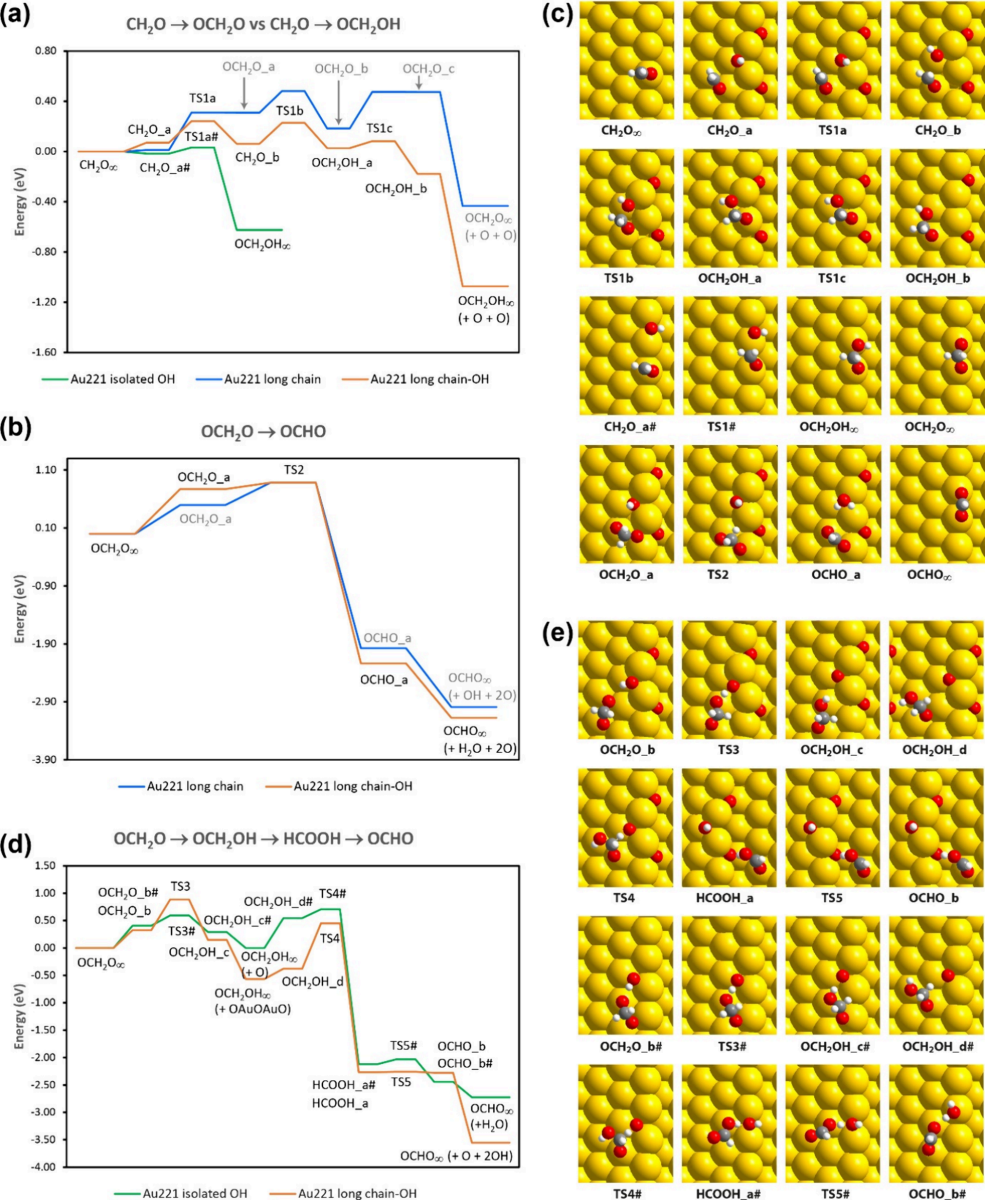
Computed reaction pathways for overoxidation of formaldehyde on the hydroxylated Au(221) surface. The relevant pathways on the non-hydroxylated Au(221) surface with a long OAuOAuO chain are also shown for comparison. Letters next to chemical names indicate different coadsorption states of CH_2_O, OCH_2_O, OCH_2_OH, HCOOH, and OCHO. The infinity symbol (∞) designates the limit of low coverage (= no coadsorbates). The hash symbol (#) indicates the pathway with isolated OH groups. Reaction energy diagram for (a) CH_2_O addition to nonterminal O or OH within an oxygen chain forming OCH_2_O or OCH_2_OH, respectively, and (b) for the H-abstraction reaction from OCH_2_O by nonterminal O or OH within an oxygen chain forming OCHO (formate). The results for reactions with the OAuOAuO chain on a non-hydroxylated surface are from our earlier work.^49^ (c) Intermediate structures corresponding to the OH pathway (orange trace) in (a) and (b). See ref (49) for the structures corresponding to the O pathway. (d) Reaction energy diagram for the formate formation from OCH_2_O via HCOOH (formic acid). OCH_2_O reacts either with an isolated OH or within an OH group within an oxygen chain. (e) Intermediate structures corresponding to the pathways in (d).

To obtain a microscopic understanding of the water modification of oxygen species at the steps, theoretical calculations were performed. The model surface chosen was Au(221), which exhibits (111) terraces separated by monatomic steps similar to Au(332). However, the terrace width and thus the unit cell are smaller compared to Au(332), which reduces the computational time. First, DFT calculations were performed to assess how hydroxylation of AuO chains affects the activation barriers for overoxidation of formaldehyde to formate, as lower barriers for hydroxylated chains can be a potential explanation for increased formate formation under wet conditions. Specifically, we focused on the barriers for reaction with the middle oxygen atom of an O–Au–O–Au–O chain, which exhibited significantly higher barriers to formate under dry conditions than single oxygen atoms or terminal oxygen atoms of AuO chains.^49^ We expect that after the formation of long O chains, the concentration of the more reactive single oxygen atoms or terminal oxygen atoms will be rather low. Consequently, the reactivity will be limited by the availability of the oxygen atoms in the middle of the extended AuO chains, which constitute a large fraction of all available oxygen on the surface. For comparison, the first part of the reaction pathway (CH_2_O → OCH_2_O or CH_2_O → OCH_2_OH) with terminal O or OH of a chain, respectively, is given in Figure S5. The calculated relative energies of all intermediates and transition states are summarized in Table S1, and the energy diagrams and the corresponding geometries are shown in Figure 7 and in Figures S5 and S10.

Note that we have calculated the reaction energies for overoxidation of formaldehyde (Figure 7) with respect to the reactants at infinite distance (indicated by a ∞ symbol in Figure 7). This approach allows for a rigorous comparison of the surfaces but corresponds to a low-coverage situation, so that the reaction energies for coadsorbed reactants are also given. For example, CH_2_O_a denotes a coadsorbed state of CH_2_O and an oxygen chain (shown in Figure 7c), which lies 0.07 eV higher than the sum of the energies of these two intermediates calculated separately (CH_2_O_∞_). A coadsorbed state often has a higher energy than the sum of the energies of isolated adsorbates not only due to steric repulsion but also because the adsorption sites and orientations of the species in a coadsorbed state may be quite different from the most favorable ones. Note also that the two OCH_2_OH_*∞*_ intermediates in Figure 7a have considerably different energies, because we are comparing two different surface reactions: CH_2_O + OH → OCH_2_OH in the green pathway and CH_2_O + OAuOHAuO → OCH_2_OH + 2 O in the orange pathway. In the first case, the reacting OH group is isolated, whereas in the second case, it is part of an oxygen chain. Similarly, in Figure 7b and 7d, OCHO_*∞*_ and OCH_2_OH_*∞*_ of different reaction pathways have different relative energies because they refer to the products of related but different reactions.

Upon hydroxylation, the barrier for the first (addition) step that converts CH_2_O to OCH_2_OH for this middle oxygen atom indeed decreases notably (from ∼0.5 to ∼0.25 eV at low coverage, Figure 7a, compare blue and orange traces). Furthermore, this step is significantly more exothermic (by ∼0.65 eV) than the corresponding CH_2_O to OCH_2_O step under dry conditions. A green trace in Figure 7a shows an analogous transformation by CH_2_O reaction with an isolated OH group, as the AIMD simulations discussed below indicate that isolated OH groups are expected to form on the surface by deprotonation of water, in addition to OH groups attached to AuO chains. In this case, the reaction is predicted to proceed with only ∼0.05 eV barrier, which is even lower than the barrier found for the analogous reaction with an isolated oxygen atom, 0.15 eV at low coverage,^49^ in rough agreement with values calculated for Au(111).^17^ Thus, the barrier for this first reaction toward overoxidation is similarly low for isolated OH or O species. However, hydroxylation of oxygen inside AuO chains lowers the barrier significantly compared to the nonhydroxylated AuO chain and thereby could contribute to increased formate accumulation. Thus, in the presence of water, hydroxylation of less reactive AuO chains but also reversible formation of isolated OH species, which are more mobile than O atoms, would yield reactive OH species contributing to increased overoxidation on the stepped gold surface.

The direct conversion of OCH_2_O to OCHO (formate), in contrast, is characterized by comparable energetics for the hydroxylated and nonhydroxylated chain (Figure 7b). This reaction step exhibits a high barrier for both hydroxylated and nonhydroxylated AuO chains, suggesting rather slow formate formation on the stepped surface for both dry and wet conditions. However, the efficient formation of the OCH_2_OH intermediate on the wet surface may not only lead to the emergence of pathways to alternative overoxidation products, such as formic acid or methanediol, but also open up an additional path for formate formation via formic acid (HCOOH), as shown in Figure 7d. The OCH_2_OH species can be formed either by the addition of OH to formaldehyde, as discussed above (Figure 7a), or by the hydrogen transfer reaction from OH to OCH_2_O converting OH to O (see Figure 7d). The latter scenario has a relatively high barrier if the OH group is within an AuO chain (close to 0.9 eV at low coverage and ∼0.6 eV from the coadsorbed state), but if OCH_2_O abstracts H from an isolated OH group on the surface, the barrier to forming OCH_2_OH is only ∼0.7 eV at low coverage and is less than 0.2 eV from the coadsorbed state. Next, OCH_2_OH can be converted to HCOOH (formic acid) by H atom abstraction from the CH_2_ group. This can be accomplished by a reaction of OCH_2_OH with O or OH. We have considered only the O case (Figure 7d), assuming that O atoms are more reactive than OH and therefore should yield lower barriers. If the reacting O atom is inside an oxygen chain, this process has a barrier of ∼1 eV under low coverage and 0.8 eV from a coadsorbed state (which is slightly higher than the OCH_2_O to OCHO barrier under dry conditions, Figure 7a). However, if the reacting O atom is isolated (it could be the same O atom formed from an OH group in the previous step, OCH_2_O + OH → OCH_2_OH + O), the barrier to formic acid formation decreases to 0.7 eV under low coverage and only ∼0.2 eV from the coadsorbed state. Although the overall energy diagram shows that TS4# for the isolated OH pathway (green trace in Figure 7d) is higher in energy than TS4 for the long-chain–OH pathway (orange trace in Figure 7d), the corresponding reaction barrier is lower for the isolated OH pathway due to a higher energy of the preceding adsorption state. Furthermore, TS4# of the isolated OH pathway (the highest barrier in this reaction path) is lower in energy than TS3 of the long-chain–OH pathway (the highest barrier in this alternative reaction path), suggesting that formation of the HCOOH intermediate is overall less activated for reactions with isolated OH/O species. The final step is OCHO formation from HCOOH, which proceeds with almost no barrier with O or OH as the H-abstracting base. Thus, in the presence of isolated OH groups at high coverage conditions, this pathway via formic acid formation may enhance formate accumulation.

These results show that hydroxylation of AuO chains and the presence of isolated OH groups on the wet surface may yield more reactive species for formate formation. This is consistent with the experimentally observed increased formate accumulation and decreased selectivity to MeFo on stepped Au(332).

### Computational Modeling of Oxygen-Covered Stepped Au(221) in the Presence of Water

**Figure 8:**
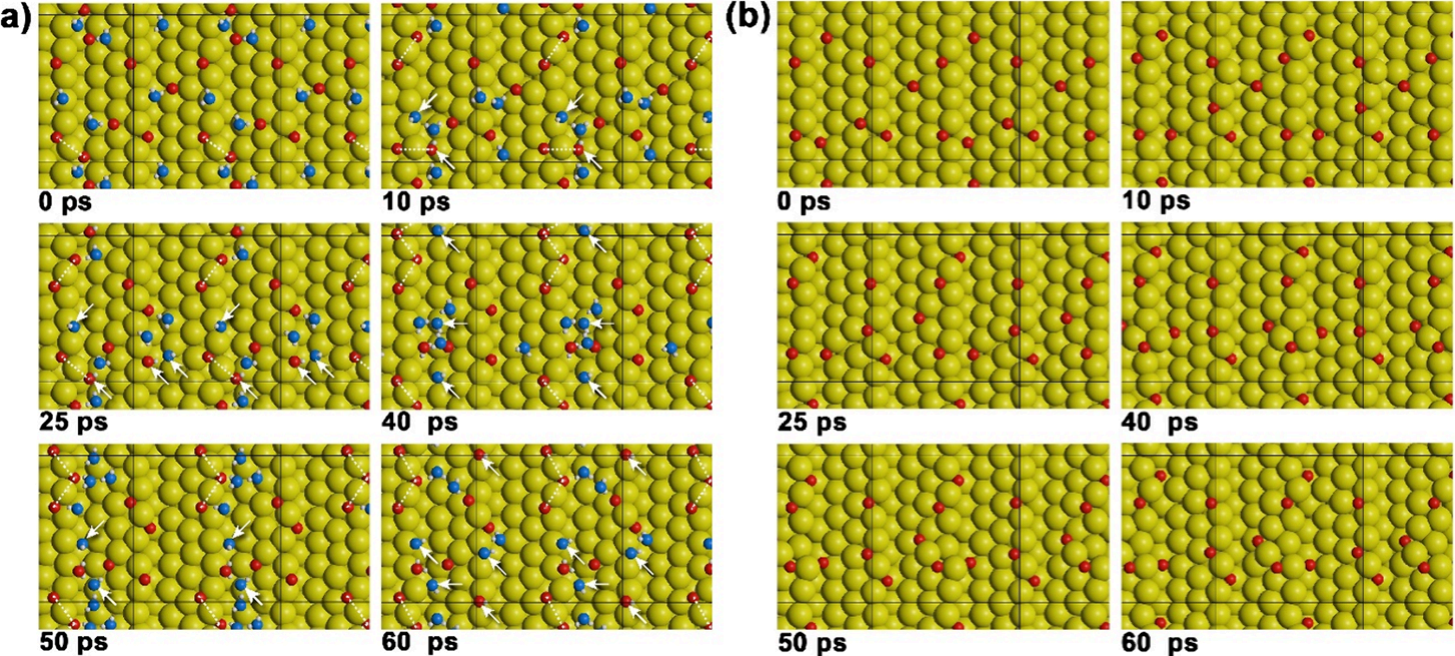
Snapshots from AIMD simulations of oxygen adsorbed on stepped Au(221) (a) with and (b) without added water. A blue and red color coding was chosen for oxygen to allow for an easier distinction between oxygen directly placed as atoms (red) and oxygen placed as part of water molecules (blue) onto the surface at the beginning of the simulation. White arrows point to OH species. Dashed lines show the oxygen chains formed.

As DFT calculations indicated that formation of different types of hydroxyl species on the stepped surface may affect selectivity in methanol oxidation, AIMD simulations were conducted to investigate how water affects the oxygen species on the stepped surface and if these different hydroxyl species are likely to be formed in the presence of water. For oxygen on stepped Au(221) under dry conditions, previous AIMD simulations showed surface restructuring coinciding with the formation of extended AuO chains.^49^ In addition to the increased barriers of AuO chains to unwanted overoxidation, we have recently provided evidence for a link between surface restructuring and MeFo formation, as efficient MeFo formation requires the emergence of highly reactive sites on the Au surfaces.^46^ Since the presence of water alters both MeFo and formate formation on stepped Au(332) during the extended pulse sequence, AIMD simulations were performed on oxygen-covered Au(221) in the presence of added water (Figure 8a) and in the absence of water (Figure 8b) under otherwise identical conditions to investigate its effect on the surface restructuring and AuO chain formation (Figure 8).

In the absence of water, the oxygen-covered Au(221) surface initially forms short O–Au–O chains, which are precursors to the longer AuO chains (Figure 8b). The formation of the longer AuO chains coincides with a considerable restructuring of the steps of the Au(221) surface.^46,49^ Isolated oxygen atoms are typically adsorbed in threefold hollow sites. In the AuO chains, the oxygen exhibits two shorter bonds and one longer bond to the Au atoms of the surface adopting thus, rather a pseudo threefold hollow or pseudo bridge adsorption geometry.^46,49^ In the presence of water, hydroxyl species are formed (Figure 8a). However, a significant fraction of the oxygen atoms does not react with water to form hydroxyls, even when a number of water molecules are in close spatial proximity to oxygen atoms. Consequently, apart from some hydroxyl species, oxygen and water also remain present on the surface under wet conditions.

In contrast to oxygen atoms, the (isolated) hydroxyls do not preferentially adsorb in (pseudo) threefold-hollow sites but are often observed in bridge-like or on-top sites (Figure 8). The hydroxyls are significantly more mobile than oxygen atoms, which show similar mobility under dry and wet conditions (see Figures S6–S8). The mobile (isolated) hydroxyls diffuse quickly over the surface, allowing them to rapidly reach more strongly binding step sites and/or adsorbed oxygen atoms to form AuO chains or extend existing chains by attaching at their ends. Terminal hydroxyls at the end of AuO chains may separate from the chain by protonation and formation of water (see Figures S9 and S10), potentially slowing the formation of longer AuO chains or leading to a breakdown of existing AuO chains in the presence of water. As illustrated in Figure S10, the activation barrier for this process (assisted by water) can be as low as 0.3 eV.

In the AIMD simulations, it is not observed that oxygen or hydroxyl attaches to the hydroxyl at the end of an AuO chain, which would lead to a continued chain growth. While isolated hydroxyls may form two or even three bonds with the gold surface atoms, hydroxyl at the end of chains is mainly observed in an on-top coordination with one bond to Au (Figure 8a), presumably due to a more covalent (or less ionic) and thus more directional bonding as compared to isolated hydroxyls. This would disfavor formation of a second bond to Au for hydroxyls at the end of chains which is required for a continuation of the “zigzag” structure of the AuO chains.

Thus, the inhibition of a direct continuation of the chain growth for hydroxyl-terminated chains suggests that the presence of water favors the growth of shorter chains. However, terminal hydroxyls may also lose the H atom via reaction with oxygen or another hydroxyl, forming a terminal oxygen at the end of the chain and allowing for further growth of the AuO chains under wet conditions. Thus, the presence of water has two competing effects: on one hand, it can slow down AuO chain growth by blocking chain ends with terminal hydroxyl groups, inhibiting chain continuation, but on the other hand, chain growth can continue under wet conditions, when terminal hydroxyls are converted to oxygen by reaction with another hydroxyl (or oxygen). The high mobility of hydroxyl species can even accelerate attachment to AuO chains, promoting their growth. Consequently, the (average) length of AuO chains formed under wet conditions will depend on the relative rates of the different reactions of the terminal hydroxyls, which in turn depends on both the relative and absolute surface concentrations of oxygen, water, and hydroxyl species. Since oxygens in the middle of longer chains were found to differ in reactivity in the methanol (over-)oxidation from single or terminal oxygens, water may affect selectivity by altering the AuO chain length and hence the relative concentrations of these oxygen species, as demonstrated by the AIMD simulations.

Since the DFT calculations suggested that hydroxylation of the middle oxygen in AuO chains affects barriers to overoxidation of formaldehyde, we investigated whether these nonterminal OH species are formed in AIMD simulations. However, no hydroxyl was found in the middle of a longer AuO chain within the simulation time. Additionally, we observed that chain growth continued only *after* conversion of a terminal hydroxyl to oxygen. No reaction of the middle oxygen atom of an O–Au–O–Au–O chain with water to form hydroxyl was detected, despite that several water molecules frequently approached this oxygen atom in the simulation. The low reactivity of nonterminal oxygen with water is presumably due to the tendency of adsorbed hydroxyls to preferentially form fewer bonds to the gold surface atoms than oxygen, making hydroxyls less favorable in chain structures. While the formation of hydroxyls in the middle of a chain cannot be completely ruled out (e.g., if nonterminal O atoms participate in H-abstraction reactions), especially as chains grow longer and terminal or single O atoms become less available, the simulations indicate that protonation of O within long chains is unlikely. If these species do form, DFT calculations indicate that they would enhance formaldehyde overoxidation on the stepped surface compared to dry conditions. More importantly, formation of isolated OH species by water deprotonation, which are more reactive than OH groups within O chains, is likely responsible for the enhanced overoxidation on the stepped surface.

Furthermore, our AIMD simulations show that the average displacement of Au atoms is lower in the presence of water and hydroxyl species, as compared to dry conditions (see also Figure S6). Consequently, the surface restructuring associated with the formation of longer AuO chains is less pronounced in the presence of water. In fact, under wet conditions, AuO chains form nearly perfectly along the steps of Au(221), unlike in dry conditions, where the steps are significantly altered by AuO chain formation. Under wet conditions, the highly mobile hydroxyls may act as oxygen carriers, facilitating AuO chain formation at the steps of Au(221) without requiring or even suppressing significant displacements of gold atoms unlike the process involving oxygen atoms. Our previous study indicated that MeFo formation on the stepped surface benefits from oxygen-induced surface restructuring, coinciding with the formation of extended AuO chains.^46^ Therefore, the suppression of surface restructuring by water may contribute to the experimentally observed decrease in MeFo formation on Au(332) under wet conditions.

Thus, the AIMD simulations provide clear evidence that the presence of water, resulting in the formation of hydroxyls that are more mobile and bind differently to the gold surface than oxygen atoms, significantly alters the formation and shape of AuO chains. Previous evidence suggested that the AuO chains are important for both high MeFo and low formate formation.^46,49^ Therefore, the AIMD simulations offer an additional reason why the selectivity of the stepped surface is altered by the presence of water. While the AIMD simulations indicate that hydroxylation of oxygen in the middle of AuO chains is unlikely, DFT calculations suggest that if it occurs, it may enhance formate formation, consistent with the experimental results obtained for the stepped surface. Additionally, isolated OH species may form in the presence of water enabling other reaction pathways, resulting in enhanced formate formation on the stepped surface under wet conditions.

As for the Au(111) surface, similar OH-related pathways should also operate there,^17^ but their importance is expected to be different compared to the stepped surface, e.g., OCHO formation by CH_2_O reactions with isolated O atoms already proceeds with comparably low or even lower barriers under dry conditions and the degree of surface restructuring by oxygen is less than on the stepped surface. Consequently, these effects of water will be less important for the selectivity of methanol oxidation on Au(111).

### Effect of Water under Methanol-Rich Conditions in MB Experiments with Extended Pulse Sequence

**Figure 9:**
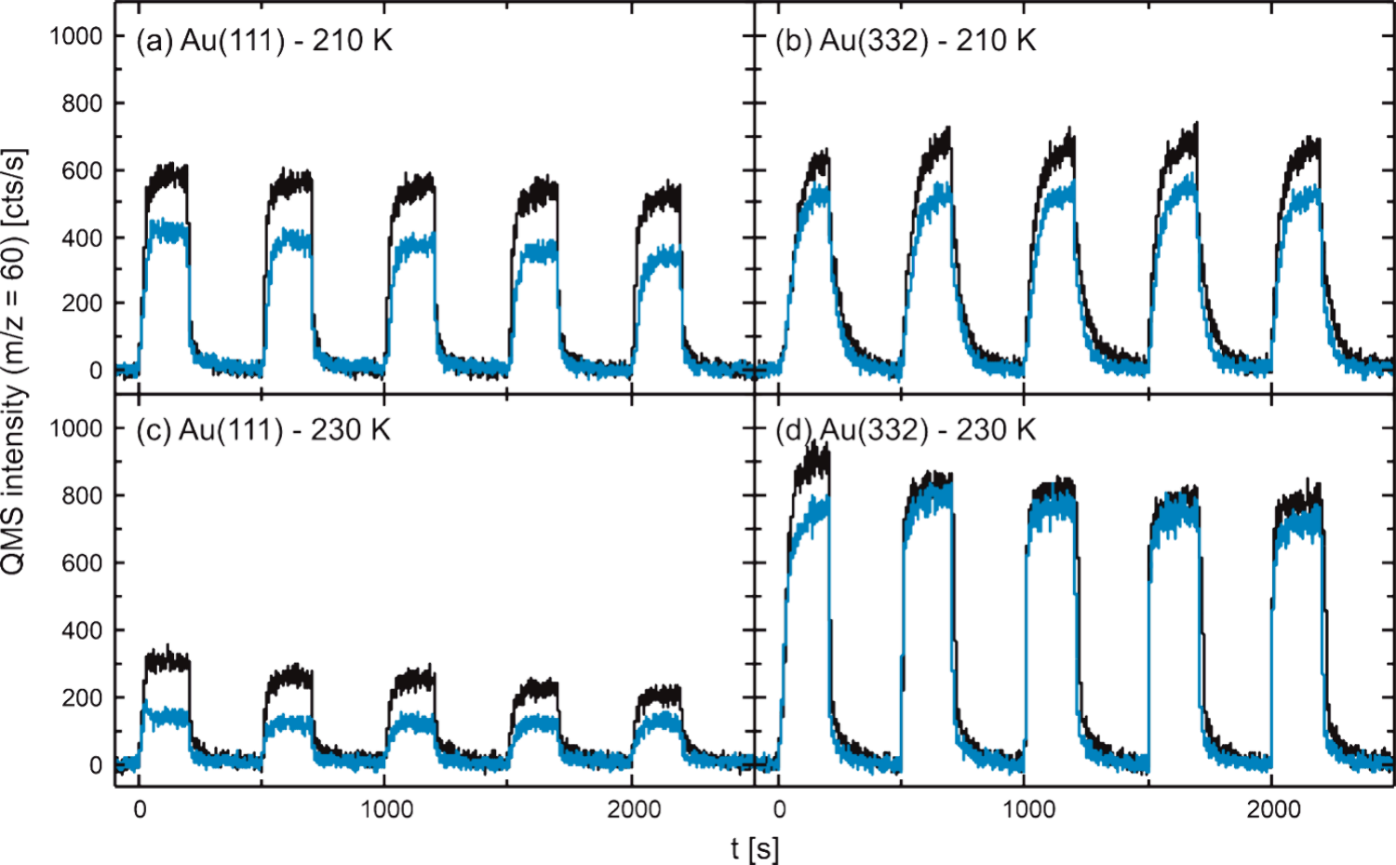
MeFo formation rate (*m*/*z* = 60) under dry (black) and wet (blue) conditions in pulsed isothermal molecular beam experiments on the methanol oxidation on (a, c) Au(111) and (b, d) Au(332) at 210 K (a, b) and 230 K (c, d). The measurements were conducted with a high continuous flux of methanol (52.7 × 10^13^ s^–1^ cm^–2^), while pulsing a low flux of atomic oxygen (0.08 × 10^13^ s^–1^ cm^–2^,5 pulses, 200 s on, 300 s off). Under wet conditions, the samples were also exposed to a continuous, high flux of water (1.6 × 10^15^ s^–1^ cm^–2^).

In the previously presented experiments with the extended pulse sequence (Figure 5), rather oxygen-rich conditions were employed, as evidenced by overoxidation to formate. In contrast, the surface concentration of activated oxygen on npAu is typically rather low.^9,51^ Therefore, we also performed experiments with a longer pulse sequence under rather methanol-rich conditions, as shown in Figure 9. For flat Au(111), water also lowers the MeFo rate under methanol-rich conditions. However, the relative rate decrease is smaller compared to oxygen-rich conditions. The decrease over the pulse sequence is comparable to (or lower than) dry conditions (see also Figure S3). Thus, water reduces the MeFo formation rate by forming less reactive OH species or by interactions with methanol but does not enhance surface deactivation under these conditions. For stepped Au(332), both the initial rate decrease and the deactivation over the pulse sequence due to water are smaller for methanol-rich conditions (Figure 9) compared to oxygen-rich conditions (Figure 5). At 230 K, the MeFo formation rate in the presence of water is essentially unchanged and constant over the pulse sequence. Thus, these results for both surfaces show not only a significantly smaller negative effect of water under methanol-rich conditions but also no water-induced deactivation over the pulse sequence under these conditions.

## Discussion

The experimental and theoretical results of this study have important implications for applied studies of methanol oxidation over npAu catalysts. In general, water has a negative effect on MeFo selectivity. The detrimental effects become strongly pronounced for conditions, where the surface concentrations of water, oxygen, and methanol become high. Such conditions are expected for the study of liquid phase methanol oxidation on npAu in a batch reactor, where the addition of relatively small amounts of water has been reported to strongly reduce MeFo selectivity.^21^ The authors suggested that the reduced selectivity may be related to the formation of a diol,^21^ and the DFT results presented here predict the formation of formic acid to compete with MeFo formation in the presence of water. Experimentally, no clear evidence of significant diol (or formic acid) formation in the presence of water was found under the conditions applied in the isothermal pulsed MB experiments (data not shown), which are expected to exhibit significantly lower surface coverages than in the liquid phase experiments. However, the detrimental effect of water on the MeFo formation becomes significantly smaller, and nearly negligible for a surface with a significant number of low-coordinated step sites, at low surface concentrations, especially those of water and oxygen. These rather low-coverage conditions are expected for gas-phase methanol oxidation on npAu in a flow reactor, typically performed at atmospheric pressure, where a high MeFo selectivity is obtained even at high conversions and correspondingly high water contents at the end of the catalyst bed.^7^ Thus, our results indicate conditions where the detrimental effect of water is nearly absent and thereby allow for understanding the seemingly contradictory results from applied npAu studies conducted in the liquid and gas phases.

In addition, our results highlight the importance of low-coordinated sites. While the beneficial effect of low-coordinated sites on undesired overoxidation under oxygen-rich conditions is lowered by water, the efficient MeFo formation at low-coordinated sites is almost unaffected by water under oxygen-poor conditions. The latter effect is attributed to altered hydrogen bonding of methanol and water at the low-coordinated step sites compared to extended terraces, while the former effect is attributed to water-induced changes in the oxygen species present on the gold surface. The AIMD simulations showed that hydroxyl formation in the presence of water affects the formation and the geometry of longer AuO chains, also reducing their stability. Since longer AuO chains exhibit higher barriers to overoxidation,^49^ this effect may, at least indirectly, increase undesired overoxidation. Furthermore, OH species may reduce the barriers to formate on a stepped surface by opening up alternative pathways through OCH_2_OH and formic acid. Moreover, water may lower the desired MeFo formation, which benefits from formation of the long AuO chains and the concomitant surface restructuring.^46^ A lower degree of restructuring of the gold surface suggests that water may also affect the coarsening of the ligaments and hence the reactivity of npAu catalysts, especially under oxygen-rich conditions.

Thus, our results enhance the microscopic understanding of the water’s role—both as a reaction product and as a common feed impurity—in the complex reaction network of methanol oxidation on gold catalysts. This improved understanding enables the identification of key conditions for applied gold catalysts to maintain high selectivity for the desired partial oxidation product.

## Conclusions

In the context of methanol oxidation on gold, we investigated the effect of water—both a product of the oxidation reaction and a potential impurity in the methanol feed—under well-defined and precisely controlled single-collision conditions. Our study involved pulsed MB experiments conducted across a range of reaction conditions to study the isothermal kinetics of methanol oxidation in the presence and absence of added water. To address the role of low-coordinated sites, we performed measurements on two types of surfaces: flat Au(111) and stepped Au(332), allowing for a direct comparison of their selectivity.

Our findings revealed a generally negative effect of water on isothermal MeFo formation, particularly when the surface concentrations of water, oxygen, and/or methanol were high, in agreement with the results for liquid phase methanol oxidation on npAu.

On the one hand, water influences methanol (or methoxy) adsorption, as evidenced by in situ IRAS. Presumably, hydrogen bonds formed with water either stabilize water itself or impact the reactivity of adsorbed methanol/methoxy, ultimately reducing MeFo formation. Interestingly, the interaction of water with methanol/methoxy adsorbed on low-coordinated step sites differs and has a less detrimental effect on MeFo formation at higher temperatures compared to (111) terrace sites.

On the other hand, water also affects the oxygen species on the Au surfaces, influencing the selectivity in methanol oxidation. The water-induced induction period for MeFo formation, associated with the formation of sites actively promoting MeFo formation and probably related to accumulated Au_*x*_O_*y*_ phases, demonstrates the impact of water in this regard. Specifically, on Au(111), undesired overoxidation to formate species decreased in the presence of water, presumably due to the formation of less reactive hydroxyl species resulting from water’s reaction with activated oxygen. In contrast, formate accumulation on the stepped Au(332) surface increased compared to dry conditions. AIMD simulations revealed that water altered the formation and geometry of extended AuO chains on steps, which lower overoxidation and enhance MeFo formation under dry conditions, compared to (111) terraces. Thus, water may counteract the beneficial effect of these low-coordinated sites.

Under low-coverage conditions, i.e., high temperatures combined with rather low methanol and oxygen fluxes, the detrimental effect of water on desired MeFo formation was mitigated. Consequently, water had a negligible effect on the stepped Au(332) surface at low oxygen fluxes. These results not only highlight the importance of low-coordinated sites for the selectivity but also provide insights into the high MeFo selectivity observed in gas-phase methanol oxidation on npAu under conditions of high conversions and therefore high water contents in the reactor bed.

Importantly, our well-defined experimental conditions shed light on how water, as a product and common feed impurity, alters the surface processes of the intricate reaction system governing selectivity in methanol oxidation on applied gold catalysts.

## Data Availability

Raw and meta data are available under DOI: 10.5281/zenodo.11067844

## Supporting Information Available

MeFo rates of isothermal MB experiments on Au(111) and Au(332) for a range of reaction conditions, in situ IRAS measurements exhibiting methanol/methoxy signals, MeFo rate decrease in isothermal MB experiments with extended pulse sequence, in situ IRAS measurements for Au(332) at 230 K for the extended pulse sequence exhibiting formate accumulation, computed reaction pathways for CH_2_O addition to non-terminal versus terminal O or OH, displacements of O and Au surface atoms on Au(221) as calculated from AIMD simulations, computed reaction pathways for the loss of the terminal OH group from an O chain, and a table of relative energies of intermediates, products and transition states along various reaction pathways (PDF)
