# Supplementary material for: Unraveling the Role of Water in Isothermal Methanol Partial Oxidation to Methyl Formate on Gold: A Combined Experimental and Computational Study
Source: J Phys Chem C Nanomater Interfaces. 2025 Jan 10;129(3):1611–26. doi: 10.1021/acs.jpcc.4c05968 (PMC11770752; doi:10.1021/acs.jpcc.4c05968)
Supplement: Supplementary file 1 — jp4c05968_si_001.pdf [file jp4c05968_si_001.pdf]

Supporting Information

Unraveling the Role of Water in Isothermal Methanol Partial Oxidation to Methyl Formate on Gold: A Combined Experimental and Computational Study

S. Eltayeb[a], L. L. Carroll[b,c], J. M. Correa-Hoyos[a], C. D. Feldt[a], B. Switon[a], W. Riedel[a], L. V. Moskaleva[b,*], T. Risse[a,*]

[a] Institut für Chemie und Biochemie, Freie Universität Berlin, Arnimallee 22, 14195 Berlin, Germany

[b] Department of Chemistry, Faculty of Natural and Agricultural Sciences, University of the Free State, PO Box 339, Bloemfontein 9300, South Africa

[c] Institute of Fundamental Physics, Consejo Superior de Investigaciones Científicas, E-28006 Madrid, Spain

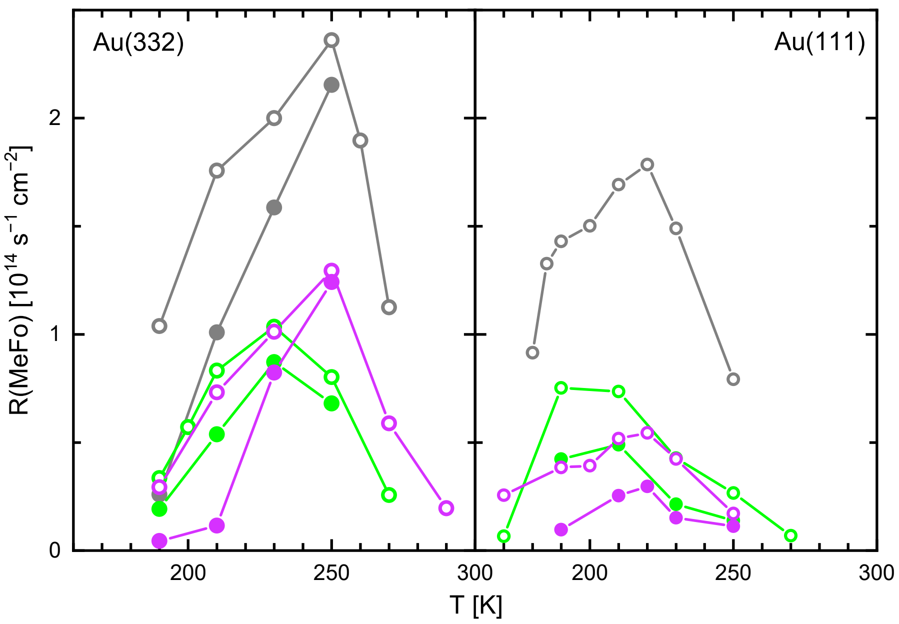

Figure S1 MeFo rate at the end of the oxygen pulse in isothermal MB experiments on Au(332) (left) and Au(111) (right) under dry (open symbols) and wet (filled symbols) conditions as a function of sample temperature, applying a constant flux of methanol and pulsing atomic oxygen (200 s on, 300 s off). An additional continuous, high flux of water (1.6×10^{15} $s^{-1}cm^{-2}$) was applied for the measurements under wet conditions. The results are shown for different methanol and oxygen flux conditions applying (grey) a high methanol flux of 52.7×10^{13} s^{-1} cm^{-2} and a rather high flux atomic oxygen of 0.4×10^{13} s^{-1} cm^{-2}, (purple) a lower methanol flux of 4.3×10^{13} s^{-1} cm^{-2}, while using the rather high flux of atomic oxygen of 0.4×10^{13} s^{-1} cm^{-2}, (green) a high methanol flux of 52.7×10^{13} s^{-1} cm^{-2}, but a lower oxygen flux of 0.08×10^{13} s^{-1} cm^{-2}.

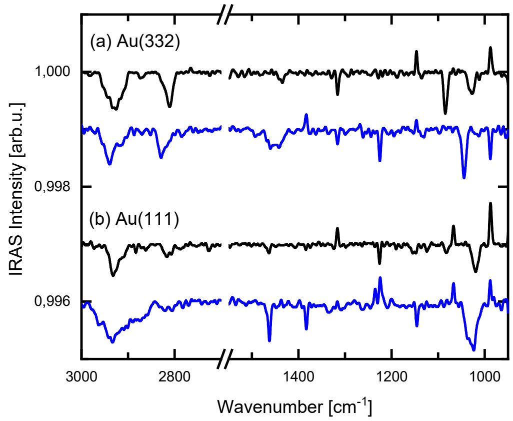

Figure S2 In-situ IRAS measurements obtained during the isothermal, pulsed MB experiments of the methanol oxidation at 190 K under dry (black) and wet (blue) conditions on (a) Au(111) and (b) Au(332). A continuous methanol flux (4.3×10^{13} s^{-1} cm^{-2}) was applied, while atomic oxygen was pulsed (0.4×10^{13} s^{-1} cm^{-2}, 200 s on, 300 s off). Under wet conditions, a continuous flux of water (1.6×10^{15} $s^{-1}cm^{-2}$) was also applied to the sample. The spectra were obtained during the oxygen pulse. The signals are attributed to methanol or methoxy species absorbed on the surface. In specific, the broad signals around 2930 cm^{-1} and 2810 cm^{-1} were previously assigned to asymmetric and symmetric C-H_3 stretching modes.[1, 2] The $\nu_{C\text{-}O}$ mode yields a sharp signal which is found under dry conditions around 1030 cm^{-1} for the stepped Au(332) surface, while it is slightly red-shifted to 1020 cm^{-1} for the flat Au(111) surface.[1, 2] Under dry conditions, the spectrum of Au(332) exhibits another signal around 1080 cm^{-1} which may be tentatively attributed to the CH_3 rocking mode of methanol/methoxy.

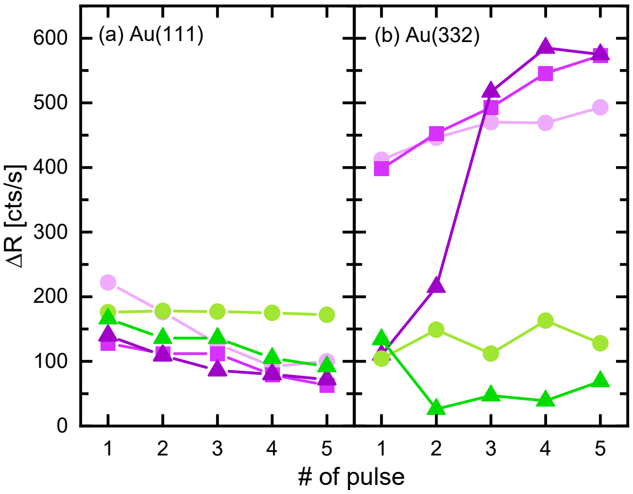

Figure S3 Absolute rate decrease $\Delta R = R_{dry}\text{-}R_{wet}$ observed under wet conditions in the isothermal MB experiments with an extended pulse sequence for (a) Au(111) and (b) Au(332). The results are shown for different surface temperatures (210 K: circles, 220 K: squares, 230 K: triangles) and two types of flux conditions: (purple) a rather low methanol flux of 4.3×10^{13} s^{-1} cm^{-2}, while using the rather high flux of atomic oxygen of 0.4×10^{13} s^{-1} cm^{-2}, (green) a high methanol flux of 52.7×10^{13} s^{-1} cm^{-2}, but a lower oxygen flux of 0.08×10^{13} s^{-1} cm^{-2}.

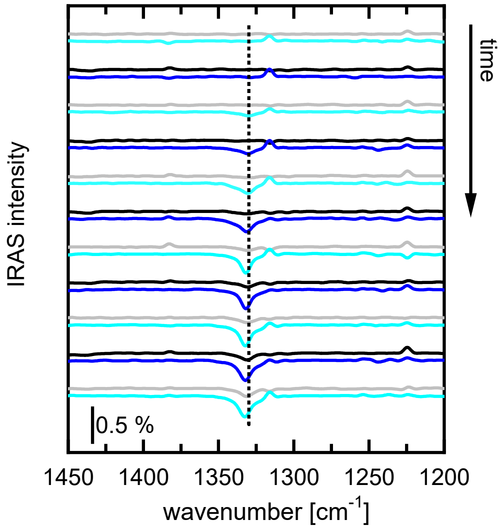

Figure S4 In-situ IRAS spectra in the wavenumber range between 1450 cm^{-1} and 1200 cm^{-1} showing the ν_s(OCO) stretching band of formate as detected in isothermal pulsed MB experiments at 230 K on Au(332) applying a methanol flux of 4.3×10^{13} $s^{-1}cm^{-2}$ and a flux of atomic oxygen of 0.4×10^{13} $s^{-1}cm^{-2}$, as well as water flux of 1.6×10^{15} $s^{-1}cm^{-2}$ under wet conditions. The in-situ IRAS measurements were conducted under dry (black, grey) and wet (blue, cyan) conditions between the oxygen pulses. The spectra were taken during (dry: black, wet: blue) and between (dry: grey; wet: cyan) the O-pulses. The top-most spectra were taken before the first oxygen pulse.

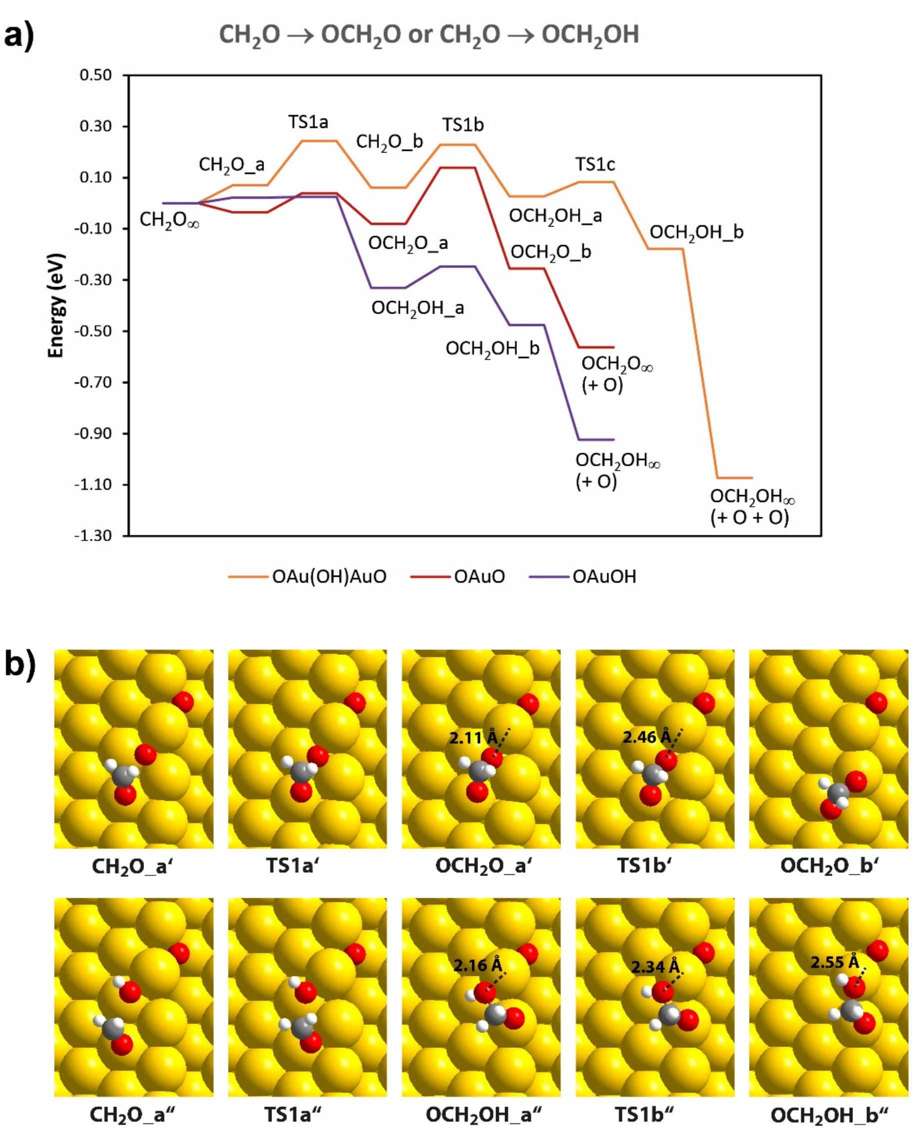

Figure S5 (a) Computed reaction pathways for CH_2O addition to non-terminal versus terminal O or OH within an oxygen chain forming OCH_2O or OCH_2OH, respectively. Letters next to chemical names indicate different co-adsorption states of CH_2O, OCH_2O, and OCH_2OH. The infinity symbol (∞) designates the limit of low coverage (= no coadsorbates). (b) Intermediate and transition state structures corresponding to the OAuO (dark red trace) and OAuOH-pathways (purple trace) in (a). The structures of the OAuO pathway are marked with the prime symbol, whereas the structures of the OAuOH pathway are marked with the double prime symbol.

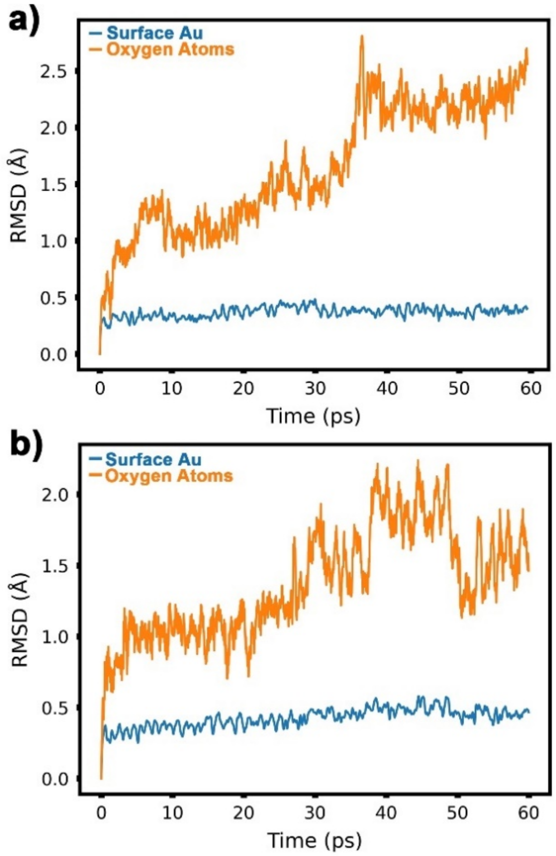

Figure S6 RMSD plots comparing the displacement of surface Au atoms and the O atoms for the Au(221) surfaces in the (a) presence and (b) absence of water. In (a) all O atoms are included that were not initially bonded to an H atom.

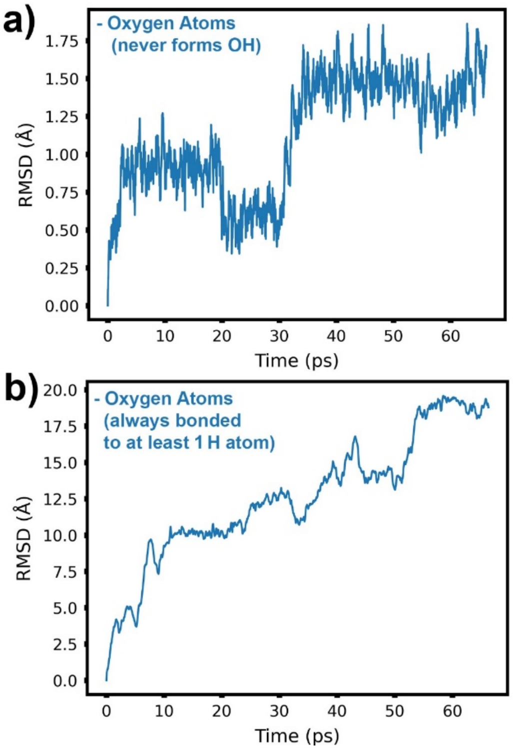

Figure S7 RMSD displacements of different O atoms in the presence of water, including water itself. (a) The RMSD of surface Au atoms and O atoms that are never within 1.2 Å from an H atom. (b) The RMSD of surface Au atoms and the O atoms that are always within 1.2 Å from an H atom.

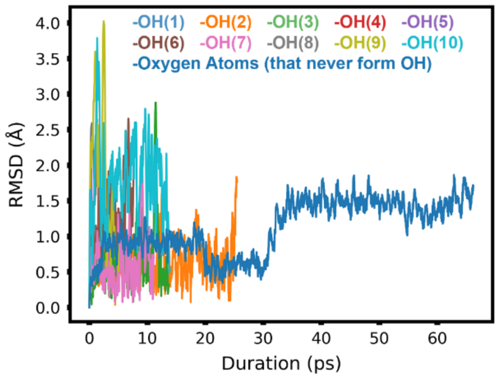

Figure S8 Duration versus RMSD plot for maximum duration exactly one OH bond is maintained for 10 different surface O atoms that ever form an OH bond, as well as the RMSD plot of the O atoms that never form OH bonds (4 of these O atoms exist). An OH bond is considered to be formed if O atom is within 1.2 Å from an H atom. Note that these RMSD values are not calculated from the first frame in the simulation, but from the first frame when the OH bond has formed, up until it is broken, or more OH bonds formed to that same O atom.

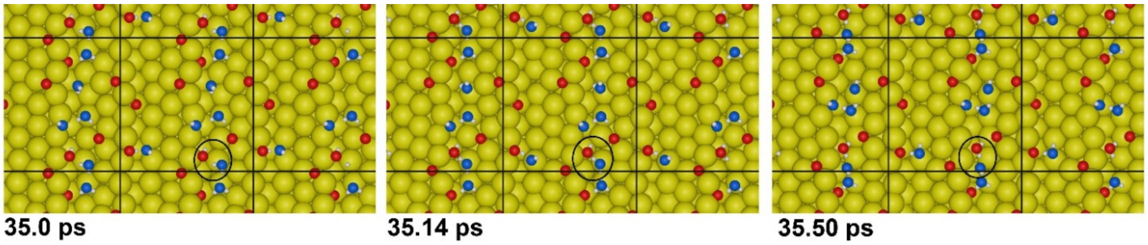

Figure S9 Proton transfer from H_2O to terminal OH of a chain for the simulation of O and water adsorbed on Au(221).

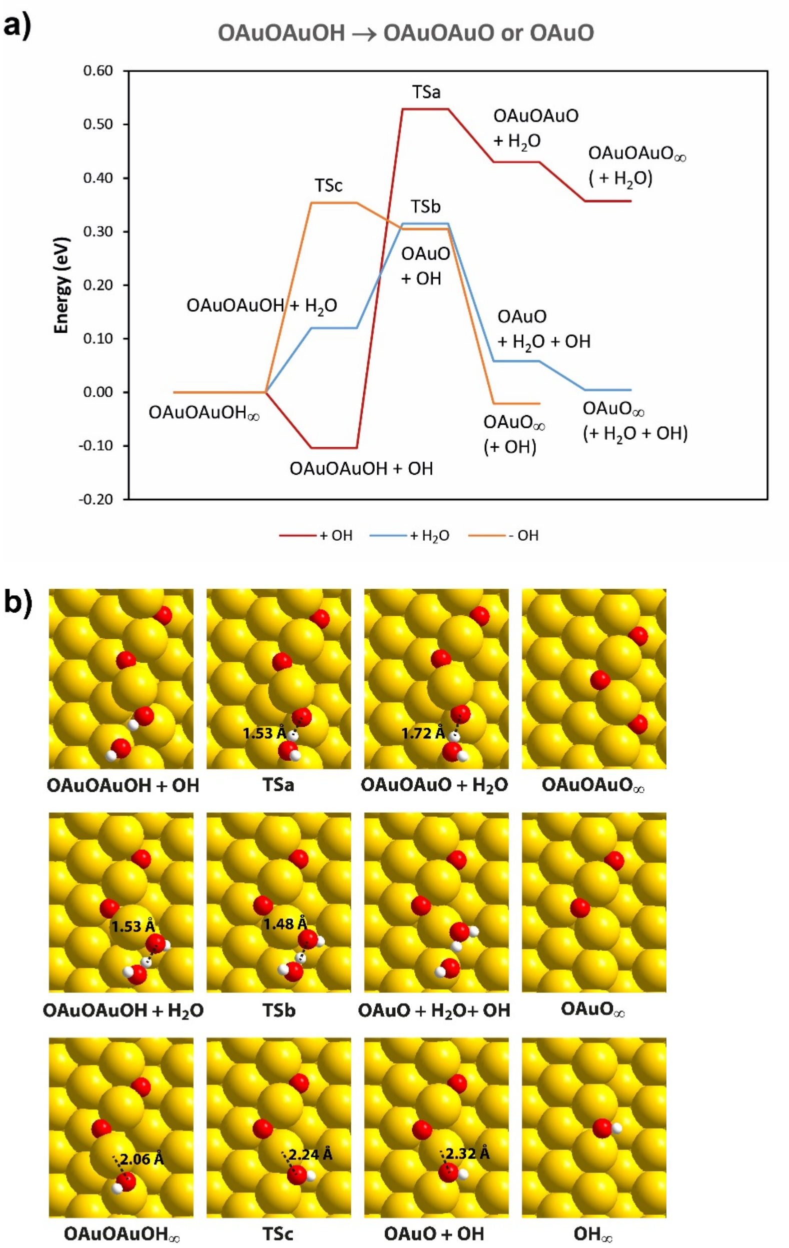

Figure S10 (a) Computed reaction pathways for the loss of the terminal OH group via its deprotonation (+ OH), protonation (+ H_2O) or dissociation (- OH). Deprotonation retains the chain length, whereas protonation and dissociation result in a loss of one O atom from the chain. The infinity symbol (∞) designates the limit of low coverage (= no coadsorbates). (b) Intermediate and transition state structures corresponding to the three pathways in (a).

Table S1 Relative energies of intermediates, products and transition states along various reaction pathways

Intermediate/ TS short name	Description	Relative Energy, eV	Intermediate/ TS short name	Description	Relative Energy, eV
**$H_2CO \rightarrow OCH_2O$ vs $H_2CO \rightarrow OCH_2OH$**					
**Long chain (OAuOAuO) Fig 7a blue trace**			**Long chain (OAuOHAuO) Fig 7a and Fig S5a orange trace**		
CH_2O_∞	CH_2O_∞ (+ OAuOAuO)	0.00	CH_2O_∞	CH_2O_∞ (+OAuOHAuO)	0.00
CH_2O_a	CH_2O + OAuOAuO	0.01	CH_2O_a	CH_2O + OAuOHAuO	0.07
TS1a		0.31	TS1a		0.24
OCH_2O_a	OCH_2O attached on both sides	0.31	CH_2O_b	OCH_2O + OAuOH + O	0.06
TS1b		0.48	TS1b		0.23
OCH_2O_b	OCH_2O attached on one side	0.18	OCH_2OH_a	OCH_2OH attached on one side	0.03
TS1c		0.47	TS1c		0.08
OCH_2O_c	OCH_2O + O + O	0.47	OCH_2OH_b	OCH_2OH + O + O	-0.18
OCH_2O_∞ (+ O + O)	OCH_2O_∞ (+ O + O)	-0.43	OCH_2OH_∞ (+ O + O)	OCH_2OH_∞ (+ O + O)	-1.07
**Short chain (OAuO) Fig S5a red trace**			**Short chain (OAuOH) Fig S5a purple trace**		
CH_2O_∞	CH_2O_∞ (+ OAuO)	0.00	CH_2O_∞	CH_2O_∞ (+ OAuOH)	0.00
CH_2O_a	CH_2O + OAuO	-0.01	CH_2O_a	CH_2O + OAuOH	0.02
TS1a		0.07	TS1a		0.02
OCH_2O_a	OCH_2O attached on one side	-0.05	OCH_2OH_a	OCH_2OH attached on one side	-0.33
TS1b		0.17	TS1b		-0.25
OCH_2O_b	OCH_2O + O	-0.23	OCH_2OH_b	OCH_2OH + O	-0.48
OCH_2O_∞ (+ O)	OCH_2O_∞ (+ O)	-0.53	OCH_2OH_∞ (+ O)	OCH_2OH_∞ (+ O)	-0.92
			**Isolated OH Fig 7a green trace**		
			CH_2O_∞	CH_2O_∞ (+ OH)	0.00
			OCH_2OH_a	CH_2O + OH	-0.02
			TS1a		0.03
			OCH_2OH_∞	OCH_2OH	-0.62

**$OCH_2O \rightarrow OCHO$**					
**Long chain (OAuOAuO)**<br>**Fig 7b blue trace**			**Long chain (OAuOHAuO)**<br>**Fig 7b orange trace**		
OCH_2O_∞	OCH_2O_∞ (+ OAuOAuO)	0.00	OCH_2O_∞	OCH_2O_∞ (+OAuOHAuO)	0.00
OCH_2O_a	OCH_2O + OAuOAuO	0.50	OCH_2O_a	OCH_2O + OAuOHAuO	0.77
TS2		0.88	TS2		0.88
OCHO_a	OCHO + OAuOHAuO	-1.98	OCHO_a	OCHO + H_2O + 2O	-2.24
$OCHO_\infty$ (+ OH + 2O)	$OCHO_\infty$ (+ OH + 2O)	-2.99	$OCHO_\infty$ (+ H_2O + 2O)	$OCHO_\infty$ (+ H_2O + 2O)	-3.18
**$OCH_2O \rightarrow OCH_2OH \rightarrow HCOOH \rightarrow OCHO$**					
**Long chain (OAuOHAuO)**<br>**Fig 7d orange trace**			**Isolated OH**<br>**Fig 7d green trace**		
OCH_2O_∞	OCH_2O_∞ (+OAuOHAuO)	0.00	OCH_2O_∞	OCH_2O_∞ (+ OH)	0.00
OCH_2O_b	OCH_2O + OAuOHAuO	0.33	OCH_2O_b	OCH_2O + OH	0.40
TS3			TS3		0.59
OCH_2OH_c	OCH_2OH + OAuOAuO		OCH_2OH_c	OCH_2OH + O	0.29
OCH_2OH_∞ (+OAuOAuO)	OCH_2OH_∞ (+ OAuOAuO)		OCH_2OH_∞ (+ O)	OCH_2OH_∞ (+ O)	0.00
OCH_2OH_d	OCH_2OH + OAuOAuO		OCH_2OH_d	OCH_2OH + O	0.54
TS4			TS4		0.71
HCOOH_a	HCOOH + OAuOHAuO		HCOOH_a	HCOOH + OH	-2.12
TS5			TS5		-2.03
OCHO_b	OCHO + OAuOHAuOH		OCHO_b	OCHO + H_2O	-2.44
$OCHO_\infty$ (+ O + 2OH)	$OCHO_\infty$ (+ O + 2OH)		$OCHO_\infty$	$OCHO_\infty$ (+ H_2O)	-2.73
**OAuOAuOH $\rightarrow$ OAuOAuO or OAuOAuOH $\rightarrow$ OAuO**					
**Deprotonation**<br>**Fig S10 red trace**			**Protonation**<br>**Fig S10 blue trace**		
$OAuOAuOH_\infty$	$OAuOAuOH_\infty$ (+ OH)	0.00	$OAuOAuOH_\infty$	$OAuOAuOH_\infty$ (+ H_2O)	0.00
OAuOAuOH + OH	OAuOAuOH + OH	-0.10	OAuOAuOH + H_2O	OAuOAuOH + H_2O	0.12
TSa		0.53	TSb		0.31
OAuOAuO + H_2O	OAuOAuO + H_2O	0.43	OAuO + H_2O + OH	OAuO + H_2O + OH	0.06
$OAuOAuO_\infty$ (+ H_2O)	$OAuOAuO_\infty$ (+ H_2O)	0.36	$OAuO_\infty$ (+ H_2O + OH)	$OAuO_\infty$ (+ H_2O + OH)	0.00

Dissociation
Fig S10 orange trace

$OAuOAuOH_{\infty}$	$OAuOAuOH_{\infty}$	0.00
TSc		0.35
OAuO	OAuO + OH	0.30
$OAuO_{\infty}$ (+ OH)	$OAuO_{\infty}$ (+ OH)	-0.02

References

1. B. J. Xu, X. Y. Liu, J. Haubrich, R. J. Madix and C. M. Friend, *Angew. Chem. Int. Ed.*, 2009, **48**, 4206-4209.
2. C. D. Feldt, T. Kirschbaum, J. L. Low, W. Riedel and T. Risse, *Catal. Sci. Technol.*, 2021, **12**, 1418-1428.
